# Pre-discharge factors predicting readmissions of psychiatric patients: a systematic review of the literature

**DOI:** 10.1186/s12888-016-1114-0

**Published:** 2016-12-16

**Authors:** V. Donisi, F. Tedeschi, K. Wahlbeck, P. Haaramo, F. Amaddeo

**Affiliations:** 1 Department of Neurosciences, Biomedicine and Movement Sciences, University of Verona, Policlinico G.B. Rossi, Piazzale L.A. Scuro 10, 37134 Verona, Italy; 2 National Institute for Health and Welfare, Mental Health Unit, Helsinki, Finland

**Keywords:** Readmission, Pre-discharge factors, Previous hospitalisations, Length of stay, Socio-demographic factors, Systematic review

## Abstract

**Background:**

Readmission rate is considered an indicator of the mental health care quality. Previous studies have examined a number of factors that are likely to influence readmission. The main objective of this systematic review is to identify the studied pre-discharge variables and describe their relevance to readmission among psychiatric patients.

**Methods:**

Studies on the association between pre-discharge variables and readmission after discharge with a main psychiatric diagnosis were searched in the bibliographic databases Ovid Medline, PsycINFO, ProQuest Health Management and OpenGrey. Relevant publications published between January 1990 and June 2014 were included. For each variable, the number of papers that considered it as a predictor of readmission and that found a significant association was recorded, together with the association direction and whether it was found respectively in bivariate and in multivariate analyses.

**Results:**

Of the 734 articles identified in the search, 58 papers were included in this review, mainly from the USA and concerning patients with severe mental disorders. Analysed variables were classified according to the following categories: patients’ demographic, social and economic characteristics; patients’ clinical characteristics; patients’ clinical history; patients’ attitude and perception; environmental, social and hospital characteristics; and admission and discharge characteristics. The most consistently significant predictor of readmission was previous hospitalisations. Many socio-demographic variables resulted as influencing readmission, but the results were not always homogeneous. Among other patients’ clinical characteristics, diagnosis and measures of functional status were the most often used variables. Among admission characteristics, length of stay was the main factor studied; however, the results were not very consistent. Other relevant aspects resulted associated with readmission, including the presence of social support, but they have been considered only in few papers. Results of quality assessment are also reported in the review. The majority of papers were not representative of the general psychiatric population discharged from an inpatient service. Almost all studies used multivariate analytical methods, i.e., confounders were controlled for, but only around 60% adjusted for previous hospitalisation, the variable most consistently considered associated to readmission in the literature.

**Conclusions:**

The results contribute to increase knowledge on pre-discharge factors that could be considered by researchers as well as by clinicians to predict and prevent readmissions of psychiatric patients. Associations are not always straightforward and interactions between factors have to be considered.

**Electronic supplementary material:**

The online version of this article (doi:10.1186/s12888-016-1114-0) contains supplementary material, which is available to authorized users.

## Background

A substantial proportion of psychiatric inpatients are readmitted after discharge. In 2011 the overall 30-day unplanned readmission rate was 13 per 100 discharged patients for schizophrenia and 11 per 100 discharged patients for bipolar disorders in 15 OECD countries. Readmissions can be disruptive for psychiatric patients and their families, and may contribute to rising costs of mental health care [1]. Readmission rates are a commonly used indicator of the quality of care and a focus of interest for all health sector policymakers [2, 3]. On the one hand, readmission rates are considered as a measure of the quality of care of the preceding hospital episode, i.e., “pre-discharge” factors are regarded as relevant, on the other hand such rates are regarded as reflecting “post-discharge” events, such as continuity of care and follow-up interventions. In psychiatry, readmission rates are widely used as a proxy for relapse or complications following an inpatient stay, indicating either premature discharge from an inpatient psychiatric ward or lack of coordination with or follow-up by outpatient facilities. Nevertheless, the associations of inpatient and community factors with readmission are far from consistent [4–7].

Among pre-discharge factors, the role of inpatient care has been less frequently assessed, with the exception of length of stay (LoS). Also, differences in ward characteristics such as the number of beds and the pressure of LoS reduction (in accordance with cost reduction) in many developed countries, have to be accounted for [2]. Finally, an extensive number of patient-based factors such as clinical and socio-demographic variables have been examined as possible direct predictors of readmission or mediators of other health process factors. Among these, if an already consistent association emerged for history of previous hospitalisations [8, 9], a weaker level of evidence was suggested for other variables [8].

The objective of this systematic review is to review and describe pre-discharge predictors of readmission after discharge from psychiatric or general health in-patient care with a psychiatric diagnosis. As far as we know, this review is the first systematic description of all the possible pre-discharge factors of readmission to hospital, reporting all the variables analysed in the literature regarding adult inpatient psychiatric populations in a comprehensive way.

## Methods

This review belongs to a series of systematic reviews from the Comparative Effectiveness Research on Psychiatric Hospitalisation by Record Linkage of Large Administrative Data Sets project (CEPHOS-LINK) on predictors of readmission. CEPHOS-LINK is a European research project investigating psychiatric services across six countries, namely Finland, Austria, Romania, Norway, Slovenia and Italy, carried out from 2014 to 2017. CEPHOS-LINK aims to compare different types of health service interventions in terms of differences in readmission outcomes in adult patients, who have been discharged from a hospital with a psychiatric diagnosis.

### Eligibility criteria

Studies on the quantitative association between pre-discharge variables and inpatient readmission after discharge for patients with a main psychiatric diagnosis were considered. The outcome of interest was readmission to inpatient hospital care, regardless of whether to a psychiatric or non-psychiatric/general bed. Admissions to day hospitals were not considered as readmissions. See Table 1 for detailed inclusion and exclusion criteria. Many outcomes on readmission are considered in the literature. In particular, the interest of this review is on the risk of being readmitted, hence only papers reporting association with readmission within a specific period from discharge as a binary outcome or as a rate were included.

**Table 1:** Inclusion and exclusion criteria, systematic review on pre-discharge factors and psychiatric readmission

	Included papers	Excluded papers
Types of studies	Quantitative studies with some quantitative measures of association between pre-discharge variables and readmission of psychiatric patients	Qualitative studies, case reports and papers not including original data, such as editorials, letters to the Editor, commentaries, reviews and meta-analyses. Studies that were not published as full reports or whose full text was not available.
Language	Papers published in English, German, Spanish, Italian and French	
Participants	Studies examining adult populations, i.e., the mean/median age of at least 18 as criterion or - when it was not possible to have direct information on that - it clearly concerned an adult population. The study participants had to be originally admitted with a psychiatric diagnosis (for example, if diagnosed using the ICD-10 system, including all diagnoses that belong to the class F00–F99 (World Health Organization, 2011)) or for a psychiatric problem (assuming this criterion as satisfied if the hospital/unit was clearly a psychiatric hospital or inpatient psychiatric unit or the authors stated that the admission episode is an acute psychiatric one).	
Outcomes		Papers reporting only analyses on other kinds of outcomes, even if connected to readmission in inpatient care (i.e., related to time to readmission or cumulative Los or number/frequency of readmissions) - results on analyses of these outcomes in the included papers were disregarded as well-.
Other exclusion criteria		The baseline did not correspond to individual patient’s discharge from hospital; it was not clear whether there was a discharge at all, or the same time-period for admissions and readmissions was considered; lack of information on the direction of any association; exclusion of readmitted patients from analysis due to modelling strategy; model either inadequate or not described; not clearly reported time of follow-up (or differing across patients with analyses not taking such variability into account); inclusion of patients dead during the index-admission among the non-readmitted; only evaluating the (comparative) efficacy of a specific drug in a trial without other predictors of interest.

### Definition of pre-discharge variables

We defined a variable as a pre-discharge predictor when it referred to the index admission period until discharge or to the period before index admission, including the discharge phase itself (for example the discharge type, discharge planning or referral decision prior to discharging the patient). In some instances, there was no clear-cut separation between pre-discharge and post-discharge variables. In the case of drug/medication interventions: if the prescription was planned or started in the pre-discharge period, it was included among pre-discharge variables, but only if the intention-to-treat criterion was adopted. Thus, such interventions were not considered in our review in case patients dropping out from the program they were assigned to were also excluded from the analyses on readmission. If a variable reflecting health system characteristics was measured at the individual level it was included in this review; on the contrary if health system variables were evaluated at aggregated level, they were described in another review of the CEPHOS-LINK project [10]. For this reason, also factors related to environmental and service characteristics are included here, as long as they are analysed at individual level. For the same reason physical comorbidity variables have been not analysed in this review [11].

### Data source and search methods for identification of studies

Comprehensive literature searches were conducted in the following electronic bibliographic databases: Ovid Medline, PsycINFO, ProQuest Health Management and OpenGrey. In addition, Google Scholar was utilized. Relevant publications published between January 1990 and June 2014 were included. No restrictions regarding publication status were used.

Studies on the association between mental health and readmission were searched using combinations of keywords (used as MeSH terms or free text, depending on the database) describing mental health services and readmission. For a more detailed description of the search terms please see Additional file 1.

### Data collection

Two pairs of researchers independently screened all abstracts (full-texts were screened, if necessary) [VD, EL and LS, RS]. Full text of all candidate papers were retrieved and screened by two researchers [VD, FT]. Discrepancies were resolved by discussion.

Available structured data on variables associated with readmission were extracted from the studies included and entered into an evidence evaluation table by two researchers [VD, FT]. The evidence evaluation table included the following information: country, study design, intervention type (in the case of intervention studies), time to follow-up, inclusion/exclusion criteria, number of participants, gender, age distribution, diagnostic groups considered in the study, both the list of all pre-discharge variables included in the analysis and which ones were found to be significantly associated with readmission (in bivariate and multivariate analyses, respectively) and in which direction (see Additional file 2).

### Quality assessment

The selected studies were assessed for quality using a set of questions broadly based on the CONSORT criteria for intervention studies and on the STROBE criteria for observational studies [12, 13]. Each study was assessed on the following criteria: representativeness of the target population to the general psychiatric inpatient population; generalizability of the hospital or unit (mainly not diagnostically specialised); participation rate and completeness of follow-up; coverage of hospital readmissions (whether to all available facilities or only to the same hospital of index discharge); controlling for confounding factors in the statistical analyses. The tool was adapted to include an assessment of topic-specific confounders such as considering any sort of history of previous psychiatric admissions, diagnosis and other characteristics. Each study was independently assessed by two reviewers [VD, FT]. Disagreements were resolved either by consensus or by a third reviewer [PH] adjudicating in the case of ongoing disagreement.

### Data analysis

First, we conducted a preliminary synthesis of study characteristics and risk of bias as evaluated from quality assessment. Studies were then organised according to predictors. The direction of effect across studies was compared for each predictor giving emphasis to results emerged in multivariate analyses, especially in the case of variables analyzed in more than one paper, and generally specifying in the text whether results referred to bivariate or multivariate analysis. In order to further synthesise the results, groups of predictors were analysed in separated tables (Tables 3, 4, 5, 6, 7 and 8), reporting in the first column the number of studies finding significant associations over the total numbers of studies analysing that variable, and in the others the number of cases where at least one significant association was found, separately for bivariate and multivariate analyses (i.e., in case multiple multivariate analyses were performed, the association was counted when that variable emerged as significant in at least one case).

## Results

### General characteristics of the included studies

**Fig. 1:**
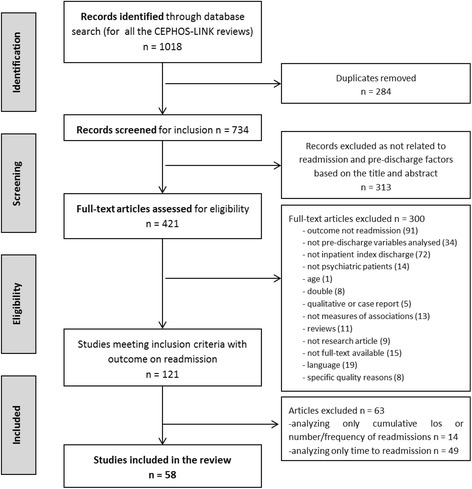
Article selection for the systematic review on pre-discharge factors and psychiatric readmission: A flow diagram. The flow-chart describes the process leading to the final selection of included papers. The global number of papers still included after each step is reported on the left, while the number of papers removed due to each exclusion criterion is reported on the right

Of the 734 unique articles identified in the search, 313 were excluded at the first stage following screening of abstracts. All other exclusions were conducted through checking full texts of the papers, and the exclusion reasons are reported in depth in the flow chart (Fig. 1). Among the 121 remaining papers, 14 had outcomes only related to number or frequency of readmissions and cumulative LoS, while 49 relating to time to readmission but not to whether patients were readmitted or not, and were thus excluded. The remaining 58 had at least one outcome relevant to this review and were hence included.

The majority of the papers included were either cohort or case-control studies, while only three were randomised control trials. In total, only seven case-control and five intervention studies were included, the remaining 46 papers being cohort studies; and among these ten were comparative (or naturalistic) studies, i.e., focusing on a single predictor.

The reviewed studies were published between 1990 and 2014, with around two thirds of the studies dating from 2000 onwards. However, the study populations included historical cohorts of patients ranging from 1984 to 2011. The majority of studies were conducted in USA (62%), five each in United Kingdom and Australia or New Zealand, two in Germany, two in China, and one each in the following countries: Malaysia, Israel, Ireland, Taiwan, Canada, Colombia, Japan, one article was a joint Egypt/Saudi Arabia study.

In terms of methodology, a comparison between readmitted versus not readmitted patients was typically performed. However, a comparison of patients readmitted before and after a given time point (i.e., “early” vs “late” readmission) was still considered among the outcomes. In particular, Priebe et al. [14] considered the readmission rate per person-year, while in other cases separate analysis were made for psychiatric versus non-psychiatric reasons [15]. Furthermore, case patients readmitted during a given time-period vs a control group of non-readmitted within a longer period [16], or early vs late readmission vs control patients [17, 18] or readmitted vs several groups of non-readmitted [19] (community and nursing home) were compared.

The follow-up period (considering the highest one in case of multiple outcomes) included a medium time-spell (between 1 month and 1 year) in around two third of cases, with 8 papers with short (up to 30 days) and 12 with long (more than 1 year) follow-up periods (see also Additional file 2 for more details on follow up).

### Representativeness, generalizability and quality assessment of papers

Results of quality assessment are reported in Table 2. The majority of papers were not representative of the general psychiatric population discharged from an inpatient service. Criteria for non-representativeness were: a particular diagnosis; studies with only or predominantly male patients or within a specific age-group (typically, the elderly); a percentage of readmitted decided by design (thus, different from the general patient population, as is typical in case-control studies); non-randomised intervention studies where controls were chosen in order to mimic the distribution of the intervention group with respect to relevant variables; choice of hospitals or patients inserted in a specific care program; specific criteria to select patients (e.g., involuntary admission or detention, first ever admission), or basing on their post-discharge planning; or specific requirements for the choice of hospital (e.g., high utilization).

**Table 2:** Number and percentage of papers basing on fulfilment of the quality criteria, systematic review on pre-discharge factors and psychiatric readmission [in brackets, corresponding reference numbers]

						Adjustment for confounding factors		
	Representativeness	Participation rate > 90%	Generalizability	Lost to follow-up < 10%	Readmission to all hospitals	previous admissions	diagnosis	other factors
Yes	15 (25.9%) [9, 43, 44, 47, 50, 53–55, 58, 62, 66, 69, 72–74]	39 (67.2%) [9, 14, 15, 20–24, 26–29, 31, 33, 34, 37, 39, 40, 43–45, 47–49, 51–55, 57–60, 62, 66, 69, 72–74]	47 (81.0%) [7, 9, 14–17, 25–39, 41–51, 53–56, 57–62, 66, 72–75]	41 (80.4%) [7, 14, 15, 18, 19, 21–25, 27, 28, 31–34, 37, 39–41, 44–54, 56, 58, 59, 61, 62, 66, 69, 72, 74, 75]	25 (43.1%) [7, 14, 15, 19, 21–24, 26, 27, 33, 35–37, 40, 43, 44, 49–52, 62, 72–74]	34 (58.6%) [9, 14–17, 19, 21–26, 29, 30, 32, 35, 39, 42, 44, 46, 48, 50–52, 54–56, 59, 60, 62, 63, 66, 69, 75]	46 (80.7%) [7, 9, 14–27, 29–35, 38–40, 42, 44, 45, 47–52, 54–56, 59–63, 66, 69, 75]	44 (75.9%) [7, 9, 14–27, 29–33, 35, 39, 40, 44–52, 54–56, 59–63, 66, 69, 75]
No	39 (67.2%) [7, 14–28, 30, 32–42, 45, 48, 49, 51, 52, 56, 57, 60, 61, 63, 75]	13 (22.4%) [16, 17, 19, 30, 32, 35, 36, 38, 42, 46, 50, 56, 61]	11 (18.9%) [18–24, 40, 52, 63, 69]	10 (19.6%) [17, 35, 36, 38, 42, 43, 55, 60, 63]	21 (36.2%) [9, 16, 18, 30–32, 38, 39, 42, 47, 53–60, 66, 69, 75]	23 (39.65%) [7, 18, 20, 27, 28, 33, 34, 36–38, 40, 41, 43, 45, 47, 49, 53, 57, 58, 61, 72–74]	12 (19.3%) [28, 36, 37, 41, 43, 46, 53, 57, 58, 72–74]	14 (24.1%) [28, 34, 36–38, 41–43, 53, 57, 58, 72–74]
unclear	4 (6.9%) [29, 31, 46, 59]	6 (10.3%) [7, 18, 25, 41, 63, 75]	0	Not applicable in 7 cases [9, 16, 20, 26, 29, 30, 57], as case-control study	12 (20.7%) [17, 20, 25, 28, 29, 34, 41, 45, 46, 48, 61, 63]	1 (1.7%) [31]	0	0

The majority of studies included both genders, apart from two which included only males [16, 20] and other seven studies were on mostly male veterans (as were the ones on only male patients) [21–27], and Zeff et al. [28] on active duty patients. In three cases [29–31] information on the gender composition of patients was not reported. In most of the studies no psychiatric diagnoses were explicitly excluded or more than one diagnostic group (in the majority of cases severe mental disorder such as psychosis or affective disorder) was considered; in a few of these studies we had to assume that an index admission to a psychiatric hospital or inpatient psychiatric unit implied a psychiatric diagnosis, as they were not reported in more detail. Some studies focused on at least one substance use disorder (alcohol dependence, alcohol abuse, alcoholic psychosis, drug dependence, drug abuse, drug psychosis) or inpatients in a substance abuse treatment program with a main alcohol/drug diagnosis or dual diagnosis patients [15, 21–24, 32]. Five studies were restricted to diagnosis of schizophrenia or schizoaffective disorders or psychosis [33–37]; four to diagnosis of affective disorder [19, 26, 38, 39]; two to diagnosis of dementia [18, 40].

The majority of papers considered all ages from 18 years old onward, in many cases until 65 even if sometimes no lower limit was explicitly reported but the service analysed was aimed to adult patients. A huge variation in the mean age (when reported) could be noticed across papers resulting in the range 25–55 years, excluding the papers focusing on the late middle age or older [15, 18–21, 40].

Most of the papers reported a participation rate over 90% of the selected population. This is due to the fact that the majority of studies used data in administrative databases or medical records. For the same reason very few papers reported a percentage of patients lost at follow-up higher than 10%.

Nearly all the datasets were from general psychiatric hospitals or inpatient psychiatric units in a general hospital (also depending on the organization of the health system in each country) and in only few papers the studied settings were diagnostically specialised units. As many studies were from USA, it is also important to note that in many papers the setting was general Veteran Affairs (VA) (psychiatric) hospitals. In around half of the papers the analysis considered readmission to all possible hospitals. In three cases, readmission was restricted to involuntary readmission in the context of involuntary index admission or detention [14, 41, 42] while in other three [33, 34, 43] involuntary readmitted patients were explicitly excluded from the analysis. In one paper readmission specific for self-harm was considered [44].

Almost all studies used multivariate analytical methods, i.e., the association between predictors and readmission was assessed controlling for confounders, but only around 60% adjusted for previous hospitalisation. In some papers [15, 34, 44–47], sociodemographic and clinical factors were just controlled for in the analyses as confounders, without showing related results.

## Categories of pre-discharge variables analysed

The pre-discharge variables analysed were classified into the following six categories: 1) patients’ demographic, social and economic characteristics; 2) patients’ clinical characteristics; 3) patients’ clinical history; 4) patients’ attitude and perception; 5) environmental, social and hospital characteristics; and 6) admission and discharge characteristics. The sections below report the results for each of these groups of variables.

### Patients’ demographic, social and economic characteristics

Among patients’ demographic, social and economic characteristics the main results are synthetized in Table 3.

**Table 3:** Synthesis of the main significant results regarding patients’ demographic, social and economic characteristics

Variables	Number of studies resulted significant/Number of studies analysing the variable	Main significant results^a^ (bivariate)	Main significant results^a^ (multivariate)
Age	15/44	Mixed direction (10)	Older age protective (8)^b^
Gender	13/46	Mixed direction (10)	Mixed direction (8)
Marital status	9/28	Being married protective factor (5)	Being married protective factor (5)
Living situation/number of cohabitant/residential stability	5/20	Mixed direction (4) Homelessness risk factor (1)	Mixed direction (4)
Education level	4/14	Mixed direction (3)	For involuntary hospitalization: education protective factor (1)
Employment status	5/15	Unemployment risk factor (5)	No significant results
Ethnical group/immigration status	6/29	Being black risk factor (2)	Mixed direction (6)
Financial status	1/6	Higher financial means protective factor (1)	Higher financial means protective factor (1)
Receiving benefits (pension or for a service-connected disability or other welfare benefits)	5/6	Receiving benefits risk factor (3)^c^	Service-connected disability risk factor (1)
Forensic and violence issues	1/3	Violence history protective factor (1)	No significant results
Military situation	1/2	No significant results	Non-service connected disability and highest income or a non-veteran protective factor (1)

^a^The number of significant results (when present) is reported in brackets for each variable. Please note that such numbers refer to the papers, and that more than one variable in the same row could be analysed in the same paper; moreover, not all studies conduct both bivariate and multivariate analysis

“Mixed direction” means that the variable resulted significant in more than one paper, but the results were contrasting

^b^plus two cases of not monotonic direction; ^c^plus one case with contrary result

In eight cases [15, 16, 21, 33, 48–51], risk of readmission was associated with younger *age* at multivariate level, but only in four cases a significant decrease in risk was found with age in all the analyses performed. However, some occurrences of non-monotonic behaviour (two at multivariate level) emerged [24, 26, 52, 53] and a higher risk for older age was found but, when also multivariate analysis was performed, in no case age remained significant [23, 34, 47].

As for *gender*, in multivariate analysis, a consistently higher risk for men resulted in four papers [31, 40, 52, 54], while, in four cases [9, 15, 55, 56] a higher risk for female patients was found.

Concerning *marital status*, being married (including also cohabitee/partner in a few studies) proved somehow protective in nine papers [21, 23–25, 33, 48, 51, 57, 58] (in four cases only in bivariate analysis). In Wong and Chung [48], the result actually just pointed out an increase in the risk for singles (but only in bivariate analysis), while in Bernardo et al. [58] and Grinshpoon et al. [51] (in the case of affective but not in that of schizophrenic patients) for divorced people.

As for *living situation*, in terms of place (mainly, whether owning a home, living in an institution or being homeless), and of household composition (i.e., with whom the patient is living, especially whether alone or not), most of the papers analysing such variables did not meet statistical significance. Living in care (vs alone or with family) was found as a protective factor in Dixon et al. [30], and Russo et al. [59] found homelessness as a risk factor at bivariate level, while living alone was found as protective in Priebe [14] and in Adams [60]. In Ono et al. [18], the variable “number of cohabitants” was considered, a larger number turning out to be a protective factor for readmission.

At bivariate level two articles on all patients with psychiatric disorders found a protective role for higher *education* (i.e., a higher risk of readmission for patients with primary education or illiteracy, and lower for those with university degree [57]; a low level of education turned out as a risk-increasing factor [36]), while one paper found a lower educational level as a protective factor for readmission [58]. The only significant association found in multivariate analysis (in one paper for subjects who were hospitalized involuntarily) highlighted that the number of years of education was associated with a decrease in readmission risk [61].

A protective behaviour of *employment* was found in five papers, but only in bivariate analysis. Being a skilled worker turned out to be a protective factor while being unemployed a risk factor [57]; full-time employment turned out as protective as well vs part-time employment, receiving social assistance or being unemployed [58]. Patients who were either employed or students showed a lower readmission risk [46]; also an increased risk was found for patients not in employment vs those who were employed (including subsistence and in the Army forces) [60] and unemployment was found as a risk factor for early readmission [29].

As for *ethnical group*, being black was found to be significantly associated with a higher risk of readmission in two papers in multivariate analyses: when examining the 5-year readmission risk (vs white patients) [26] and the 60-day readmission risk (vs native American and Asian patients, only for some subgroups analysed) [49]. In Phibbs et al. [24], on the contrary, being black (vs white) turned out as a protective factor. The other ethnical group meeting significant results in the literature was the Hispanic one, associated with a lower risk of readmission at 8–30 days (compared with white patients) in Mark et al. [52], but with a higher risk (compared with white and other non-black patients) in Stahler et al. [32] and (compared with white and black patients) in Becker and Shafer [33].

Among *socioeconomic factors*, income, socioeconomic status and financial status were not significantly associated to readmission in five papers [19, 25, 28, 48, 55], while higher financial means were found as a protective factor in Owen et al. [62]. The variable “presence of a disability support pension” resulted as a risk factor (only in bivariate association) in Callaly et al. [29], as well as being in receipt of welfare benefits in Priebe et al. [14]. In Phibbs et al. [24] service-connected disability turned out as a risk factor at multivariate level, while contrasting results emerged at bivariate level [21, 26].

Finally, variables related to forensic and violence issues were analysed in three papers, but only in Wong and Chung [48] violence history was associated with a decreased risk of readmission (only at bivariate level). Other variables related to *military service* were analysed (years of active duty service, branch of service, military rank), but only a composite indicator - being either a “means-test C” (i.e., non-service connected disability and highest income) or a non-veteran - was found as a protective factor [24].

### Patients’ clinical characteristics

*Diagnosis*, defined as primary psychiatric diagnosis, was the main clinical characteristic of the patients analysed, but different grouping methods were adopted trough the papers. Results turned out to be not significant in 18 cases. Due to the large amount of information, only the main significant results reported in multivariate analysis are presented in the text. Having a psychotic disorder resulted in an increased risk to being readmitted in two papers [52, 56], having a mood disorder or a substance abuse diagnosis in one [52], and personality disorder in one paper [54]. In Swartz et al. [61], having psychosis compared with affective disorders resulted in a decreased risk of readmission only for one of the two sub-groups of patients being discharged to an outpatient commitment group. In Sanchez et al. [55], having a secondary psychiatric diagnosis (the primary being a medical condition) was a protective factor compared with having bipolar disorder as the primary diagnosis. Among severe mental disorders, in Thompson et al. [63] schizo-affective disorders increased the risk compared with other schizophrenic disorders.

When explicitly examined, the presence of a secondary diagnosis of substance abuse or dependence (or substance abuse complications) resulted in an increase of the risk of readmission in some multivariate analysis [52, 55, 59], while decreasing risk in one study [50]. Substance abuse patients with mental and behavioural disorders due to psychoactive substance use were more likely to be readmitted [15, 21–24]. Moreover, in Phibbs et al. [24], differences among type of substance of abuse emerged and in Kim et al. [26], a major depressive disorder diagnosis (versus “other depression diagnosis”) and a tobacco use disorder were negatively associated with hospital readmission.

Finally, psychiatric comorbidity with other psychiatric diagnoses was also explicitly examined with non-homogeneous results. Number of psychiatric diagnoses was significant in one paper [15]. Presence of a personality disorder when resulted significant increased the risk of readmission at multivariate level in [9, 33]. A study by Stahler et al. [32] found that having a chief complaint of depression decreased the risk of readmission among patients with dual diagnosis.

*Physical comorbidity* has been studied as possible predictor as well: results have been reported in another review of the CEPHOS-LINK project [11].

In terms of *suicide*, in Lyons [7] suicide potential as a reason of admission decreased the risk of readmission at 1 year, but not at 30 days or at 6 months. In Kim et al. [26] a history of suicide attempt increased the risk of readmission in one paper at bivariate analysis, but resulted not significant in other two papers [48, 58]. In Monnelly [16], when at least a sign of instability during hospitalisation was reported, the risk of readmission increased although suicide alone was found not significant. Finally, in Wong and Chung [48] family history of suicide seemed to make this group of patients more vulnerable, indicating a relatively higher risk of readmission in bivariate analysis because of further mental deterioration provoked by this social stress.

Lower Global Assessment of Functioning (*GAF*) [64] scores resulted in an increase of the risk of readmission as measured at admission ([25, 43] - at bivariate level; [50] – at multivariate level) and in the previous 4 months before admission [61]; and in one paper [16] (at the bivariate level) when GAF was measured at discharge. When previous GAF was evaluated significance was found for the lowest value in the prior year (only in bivariate analysis) [25]. A greater severity corresponded to a lower risk of readmission, but only when comparing readmission vs nursing home disposition, while no significant differences emerged between readmission to hospital and continuous stay in the community [19]. Patient clinical status was also analysed through *other scales of functioning or psychopathology*, together with *measures of cognitive status*, *quality of life*, *psychosocial problems or history of behavioural problems* (e.g., aggression). At least one significant association with readmission was found in 12 papers (in four papers only at bivariate level [9, 19, 58, 62]). Few studies used different versions of the Brief Psychiatric Rating Scale (BPRS) [65]. When BPRS resulted significant, readmitted patients had a higher score on 24-item BPRS at discharge [66], but direction of the significant association resulted reversed using a 23-item version of BPRS at admission at bivariate level [59]. At multivariate level, higher scores in the anxiety index of The Symptom Checklist 90 Revised [67] and in the Behavior and Symptom Identification Scale [68] measured at hospital admission increased the risk of readmission [46, 69].

In Lyons et al. [7] using “the Severity of Psychiatric Illness scale” and “The Acuity of Psychiatric Illness scale”, the 30-day readmission risk increased for higher level of self-care impairment, 6-month readmission risk for higher clinical status scores at admission and higher level of severity of symptoms and 1-year readmission risk for self-care impairment, severity of symptoms and premorbid dysfunction level.

More psychosocial problems evaluated at discharge using DSM Axis IV [64] were found associated to readmission, but only at bivariate level [19], while one of their items (economic problems) turned out as a risk factor in multivariate analyses [49]. Other different measures of functioning resulted significant in some papers at bivariate and multivariate level. In this latter case, activity of daily living dysfunction was found as a risk factor ([50] and, for women with dementia, both at admission and at discharge [18]).

One paper [59] analysed the quality of life, finding a lower risk of psychiatric readmission for patients: with more social contacts and frequency of contacts with family (by telephone) and visits with family and with friends, with higher global life satisfaction reported both at admission (also at multivariate level) and at discharge, and with more satisfaction for each of the following subscales: living arrangements, family relations, social relations, leisure activities, personal safety, and finances.

Cognitive impairment resulted associated to readmission in patients who were hospitalised in a ward for dementia but only at bivariate analysis and in late readmission vs control or early readmission with differences between genders [18], with late readmission more likely for women and less likely for men with higher cognitive function.

In a few papers different *proxies of severity* as a subjective evaluation by staff members were analysed, resulting not significant in two papers [45, 49]. In other studies, a poor versus fair or good prognosis increased the risk of readmission [63] in multivariate analysis, as well as, at bivariate level, requiring extensive assistance [40] and (considering early vs late readmission) having any active symptomatology and affective symptoms (across all diagnoses) or presence of psychotic symptoms at discharge (only among patients with schizophrenic/schizoaffective disorders) [17].

Table 4 synthetizes the main results for this group of variables.

**Table 4:** Synthesis of the main results regarding patients’ clinical characteristics

Variables	Number of studies resulted significant/Number of studies analysing the variable	Main significant results^a^ (bivariate)	Main significant results^a^ (multivariate)
Psychiatric Diagnosis	28/46	Mixed results and different diagnostic groups compared (20)	Mixed results and different diagnostic groups compared (17)
Suicide attempt or gesture (history or risk during hospitalization)	3/6	A history of suicide attempt (1) and a family history of suicide (1) risk factors	Suicide potential protective factor (1)
GAF^b^	6/11	Measured in different moments (4)	Measured in different moments (3)
Subjective prognosis and risk score	3/5	Symptomatology at discharge (1) and patients required heavy care risk factor (1)	Poor prognosis risk factor (1)

^a^The number of significant results (when present) is reported in brackets for each variable. Please note that such numbers refer to the papers, and that more than one variable in the same row could be analysed in the same paper; moreover, not all studies conduct both bivariate and multivariate analysis

^b^See the text for other results on measures of functioning and psychopathology

Finally, antipsychotic and substance use prescription fill in 6 months before the index hospitalization resulted associated with readmission [52] as well as the number of medications filled during the year before but with a non-monotonic association [26].

### Patients’ clinical history

*Admission history* turned out to be significantly associated with readmission in 32 out of 37 studies, resulting in 31 cases as a risk factor. In 20 of these studies such relationship was found in all the multivariate analyses performed, while in one other case only in some of the different multivariate regressions performed; only in one case association was found at bivariate but not at multivariate level [66]. In just one study and only in bivariate analyses [14], a negative relationship was found between having been previously hospitalized and readmission risk.

*Duration of illness* was considered in four papers [25, 37, 55, 57]. Two papers [25, 57], found a significant association (with length of illness being a risk factor for readmission, only in bivariate analyses). In Wong and Chung [48], a decrease in the risk of readmission was found for older *age at onset*. A recent French study [38] compared three groups: late and early onset geriatric patients and young adults. In this case, late onset turned out to be a risk factor (while the lowest risk was found for young adults). In Ng et al. [66] an *index admission corresponding to the first onset of illness* was found as a protective factor towards readmission within 6 months from discharge, but only in bivariate analysis (the authors suggesting that being at the first onset was associated with a lower risk of readmission due to compliance of medication), while in another study [19] no significant association between first onset and readmission was found for older adults hospitalized for depression.

*Number of hospital days* in a given period before index admission was found associated to higher risk ([25] and, only in bivariate analysis, [26]) while, in Moos et al. [21], it turned out as non-significant. The average length of hospital stay in previous admissions was also considered in one study, turning out to be non-significantly related to readmission [48].

Several measures of *non*-*hospital pre*-*admission contacts with health services* were analysed. Being known to the mental health service before index admission [9], previous use of outpatient mental health services [23, 26, 50, 52], and preadmission relationship with a mental health practitioner [31] were found as predictors of readmission in multivariate analyses. Three papers considered outpatient medical visits before index admission [21–23]; Moos et al. [21, 23] found them to be a significant risk factor at multivariate level. Moos et al. [22, 23] also analysed at multivariate level the effect of prior inpatient treatment for a medical condition: it was associated with an increased risk of readmission in both studies.

Table 5 synthetizes the main results for this group of variables.

**Table 5:** Synthesis of the main results regarding patients’ clinical history

Variables	Number of studies resulted significant/Number of studies analysing the variable	Main significant results^a^ (bivariate)	Main significant results^a^ (multivariate)
Previous admissions	32/37	Previous admissions risk factor (23)^b^	Previous admissions risk factor (21)
Duration of illness	2/4	Higher length of illness risk factor (2)	No significant results
Age at onset	2/6	Mixed direction (2)	No significant results
Whether index admission corresponded to first onset/episode	1/2	First onset protective factor (1)	No significant results
Number of previous hospital days/average previous LoS	2/4	Number of previous hospital days risk factor (2)	Number of previous hospital days risk factor (1)
Previous use of health services	8/10	Increasing risk with service use (3)	Increasing risk with service use (8)

One paper with not significant results on age at first psychiatric admission was also found

^a^The number of significant results (when present) is reported in brackets for each variable. Please note that such numbers refer to the papers, and that more than one variable in the same row could be analysed in the same paper; moreover, not all studies conduct both bivariate and multivariate analysis

“Mixed direction” means that the variable resulted significant in more than one paper, but the results were contrasting

^b^plus one with contrary result

### Patients’ attitude and perception

Higher *patient*’*s satisfaction* on different aspects of hospital treatment decreased the risk of readmission, controlling for other variables [14]. Some studies have evaluated patient’s *attitude towards care* as possible predictors. In Kottsieper [56], both at bivariate and multivariate level, a positive attitude toward medication was found to decrease the risk of readmission, but past aftercare adherence, self-determination and internalization for motivation for psychotherapy turned out as non-significant. In Russo et al. [59] an increase of risk was found for patients with a better *insight into their psychiatric illness* at admission at multivariate level.

Table 6 synthetizes the main results for this group of variables.

**Table 6:** Synthesis of the main results regarding patients’ attitude and perception

Variables	Number of studies resulted significant/Number of studies analysing the variable	Main significant results^a^ (bivariate)	Main significant results^a^ (multivariate)
Patient’s satisfaction with treatment	1/1	Satisfaction protective factor (1)	Satisfaction protective factor (1)
Patient’s compliance, attitude toward medication and follow up visits	5/8	Positive attitudes protective factor (4)^b^	Positive attitudes protective factor (1)
Insight into illness/denial of diagnosis or prognosis	3/5	Caregiver’s denial risk factor (1) A sealing over recovery style risk for involuntary readmission (1) ﻿Insight risk factor (1)	Insight risk factor (1)

One paper with not significant results on perceived coercion and on perceived risk to self or others was also found

^a^ The number of significant results (when present) is reported in brackets for each variable. Please note that such numbers refer to the papers, and that more than one variable in the same row could be analysed in the same paper; moreover, not all studies conduct both bivariate and multivariate analysis

^b^plus one where readmission status was associated with having a greater level of intent to attend outpatient medical and psychiatric appointments

### Contextual factors: environmental, social and hospital

Environmental factors such as hospital location and variables related to neighbourhood environment characteristics, health system factors and social context factors (family and caregivers relationships) were considered in this category.

A comparison between *urban* (or metropolitan) and *rural* (or non-urban) areas was performed in five papers. An urban setting was found as a risk-increasing factor in one study [52], while a higher risk for rural areas was found in another study, where however only bivariate analysis was performed [57]. Some papers analysed differences in readmission risk related to *hospital or discharge location*, but are referred to specific national situations; in particular, Kim et al. [26] and Adams [60] compared US regions and Lin et al. [34] Taiwanese regions.

Stahler et al. [32], considered many variables related to *neighbourhood environment characteristics and services distances*, finding a higher risk of readmission for patients who lived in close proximity to a Narcotics Anonymous meeting location and a lower one for patients living in areas with higher educational attainment. *Unavailability of resources*, measured in terms of either absence of services and resources required by the patient in the geographic area to which the patient had access, or a waiting list making them non-usable, was also measured but resulted as not significant [40].

*Physician gender and experience* (using age as a proxy) were examined with bivariate analysis, gender turning out to be non-significant and experience being protective [34]. The same study analysed also *other hospital*-*level variables* and found that being discharged from medical centres or not-for-profit hospitals was a protective factor, while patients discharged from regional and public hospitals had the highest readmission rates. In Mark et al. [52], lower median length of stay and higher annual mean number of stays for Medicaid patients with mental or substance use disorder (M/SUD) or some psychiatric/psychological procedures (interviews, consultations and evaluations; somatotherapy, individual psychotherapy) turned out as risk factors and other psychiatric/psychological procedures (other psychotherapy and counselling, alcohol and drug rehabilitation and detoxification) as protective factors, with the annual mean number of stays of patients with M/SUD diagnosis and the median LoS being significant also in the multivariate analyses.

We also considered two economic issues partially related to the health system characteristics, but analysed at individual level. As for papers related to *payment*/*reimbursement mechanisms* and *insurance*, Medicaid was found as a protective factor (vs commercial insurance) in Kolbasovsky [45] while mixed results emerged in bivariate analysis [52, 56].

Among variables related to *social support*, at multivariate level, insufficient emotional and practical support of caregivers increased the risk of being readmitted [40], as well as did maladaptive family system functioning [20] and social support unreliability [50]. Also, for women with dementia, having caregivers who felt burdened by care responsibilities increased risk of late readmission versus no readmission [18].

At bivariate level, criticism of family member and caregiver’s over-estimation of their own ability to provide assistance and emotional support, more family involvement, attendance of a carer at the discharge planning, perceived treatment support reported significant results [7, 25, 35, 37, 40, 57]. On the contrary, presence and extent of social support network, pre-discharge contacts with family or non-government psychosocial support organisations, change in the support system preceding hospitalisation and family conflict resulted non-significant.

Table 7 synthetizes the main results.

**Table 7:** Synthesis of the main results regarding contextual factors: environmental, social and hospital

Variables	Number of studies resulted significant/Number of studies analysing the variable	Main significant results^a^ (bivariate)	Main significant results^a^ (multivariate)
Urban/metropolitan vs rural^b^	2/5	Mixed direction (2)	Urban residence risk factor (1)
Environmental variables, services distance and availability of resources	1/2	No significant association	Living in close proximity to a Narcotics Anonymous meeting location risk factor while living in areas with higher educational attainment protective factor (1)
Physician characteristics and other hospital-level variables	2/2	Different variables analysed and found significant (2)	Number of Medicaid patients with mental or substance use disorder (1) and shorter median LoS (1) risk factor
Fee-for-service or capitated Medicaid plan or (type of) insurance coverage	3/4	Mixed direction (2)	Medicaid (vs commercial insurance) protective factor (1)
Social support	9/14	Social support protective factor (6)^c^ (different variables analysed)	Social support protective factor (4) (different variables analysed)

^a^The number of significant results (when present) is reported in brackets for each variable. Please note that such numbers refer to the papers, and that more than one variable in the same row could be analysed in the same paper; moreover, not all studies conduct both bivariate and multivariate analysis

“Mixed direction” means that the variable resulted significant in more than one paper, but the results were contrasting

^b^See the text for differences in readmission risk related to hospital or discharge location referred to specific national situations

^c^plus 1 where readmission status was associated with having increased levels of perceived treatment support from significant others

### Admission and discharge characteristics

*Length of stay* was examined in many studies. In Ono et al. [18], higher values of LoS turned out to be a risk factor for early readmission (in the first 3 months), but a protective factor towards late readmission (from the 4th to the 24th month), such results being confirmed also in multivariate analyses. In four studies [26, 28, 46, 53], only at bivariate level, a longer LoS resulted as a risk factor of being readmitted. In four studies a longer LoS turned out as a protective factor in multivariate analysis: toward readmission at 28–30 days for patients with different psychiatric diagnoses [34, 54], and at 4–5 years for patients respectively with substance use disorders or schizophrenia [21, 33]. Moreover, a longer LoS turned out as a protective factor also in three papers only performing bivariate analysis [36, 44, 57].

The *legal status of the index admission* was considered among the potential predictors in nine papers, with a higher risk for voluntarily admitted patients found in Hendryx et al. [49] (vs court-order admitted patients) and (but only in bivariate analysis) Russo et al. [59]. In this last study, readmission rates for chronic patients assigned to a locked unit decreased.

As for *type of discharge*, escape from hospital or discharge against medical advice increased risk of readmission in two papers [30, 57] and in one study the 90-day readmission risk increased for discharge referral to other centres due to remission versus discharged on medical advice, but not for discharged against medical advice [55]. Adequacy of discharge planning (as evaluated by a social worker) turned out instead as a protective factor [40] in multivariate analyses, as well as having a discharge plan sent to the GP on discharge from the index admission [9, 29]. *Discharge destination *- planned during admission - in terms of accommodation (e.g., community centres, home) resulted non-associated to readmission, apart from being followed by the social welfare services which increased risk of readmission compared with referral to relatives [48] as well as (in bivariate analysis) having an assigned service in community [33]. Moreover, one paper reported a decreased risk for patients assigned to an outpatient (vs control) commitment group, both alone and in interaction with psychotic diagnosis [61].

*Complications during hospitalisation* for patients suffering from dementia resulted as not significant in multivariate analysis but increased risk for early readmission when looking at bivariate associations [18]. In Monnelly [16], when at least a sign of instability during hospitalisation (i.e., use of restraints, use of seclusion, requiring orders for close observation in the 3 days before discharge, active psychotic behaviour, suicide attempts or gesture, assault within 5 days of discharge, receiving p.r.n. medications - not including hypnotics - or not) was reported, the risk of readmission increased (as well as for each sign separately, apart from those occurring most rarely, i.e., suicide and assault), while in [54] admissions involving reportable aggressive incidents were found as more likely to lead to readmission (in both papers, also in multivariate analyses).

Six papers [17, 28, 35, 39, 48, 66] analysed different aspects of *pharmacological treatment* (such as dosage or medication prescription), but it resulted significant in only the three of them discussed below and always in bivariate associations. Being on depot injectable antipsychotic medication turned out as a risk factor, while using atypical antipsychotic medication was protective towards readmission [66]. Prescription of atypical antipsychotic was again found as a protective factor (while no significance was found for depot) [35]. Receiving mood stabilizers was found as a protective factor for patients with bipolar disorder, while receiving antipsychotic medications for those with depressive psychosis [17].

Intensive case management (ICM) was found as protective versus control group in multivariate analysis [45]. Other three papers analysed *other interventions during hospitalisation* finding significant results only at bivariate level: in one receiving ECT during the hospital stay reduced the risk of early readmission [19] - this variable resulted not significant in another paper [66] -, an intervention of advanced directives (a statement of a person’s preferences for treatment during admission) versus control group did not result in statistically different readmission risks [42].

Table 8 synthetizes the main results.

**Table 8:** Synthesis of the main results regarding admission and discharge characteristics

Variables	Number of studies resulted significant/Number of studies analysing the variable	Main significant results^a^ (bivariate)	Main significant results^a^ (multivariate)
Length of stay	13/33	Mixed direction (11)	Protective factor (4), mixed results (1)
Involuntary admission	2/9	Involuntary admission protective factor (2)	Involuntary admission protective factor (1)
Type of discharge	6/10	Discharge plan sent to GP (2), located (1), rated as adequate (1) and discharge on medical advice (2) protective factors	Discharge plan sent to GP (2), rated as adequate (1), discharge on medical advice (2) protective factors
Referral made at discharge/discharge destination	3/6	Being followed by social welfare services (1), having an assigned service in community (1) risk factors	Being followed by social welfare services (1) risk factor; patients assigned to an outpatient (vs control) commitment group protective factor (1)
Complications during hospitalization	3/3	Complications during hospitalization risk factor (3)	Complications during hospitalization risk factor (2)
Treatment and clinical practice	5/9	Atypical antipsychotic (2), receiving mood stabilizers at discharge (1), antipsychotic medications (1), ECT in the hospital stay (1) protective factors; on depot injectable antipsychotic (1) risk factor	Intensive case management services protective factor (1)

One paper with not significant results on “treatment goals documented at admission” and on “treatment goals met at discharge” was also found

^a^The number of significant results (when present) is reported in brackets for each variable. Please note that such numbers refer to the papers, and that more than one variable in the same row could be analysed in the same paper; moreover, not all studies conduct both bivariate and multivariate analysis

“Mixed direction” means that the variable resulted significant in more than one paper, but the results were contrasting

## Discussion

This review identified a wide range of studies on the association between pre-discharge variables and the risk of readmission. Analysed variables were classified according to the following categories: patients’ demographic, social and economic characteristics; patients’ clinical characteristics; patients’ clinical history; patients’ attitude and perception; environmental, social and hospital characteristics; and admission and discharge characteristics.

### The role of patients’ characteristics

A lot of sociodemographic and clinical characteristics of the patients have been studied as possible influencing factors of readmission. The awareness of the likely effect of these factors is useful for health professionals in order to detect high risk populations to whom possibly address prevention strategies.

Among sociodemographic variables, age and gender were analysed in most of the papers; however, turning out as non-significant in the majority of them. Although often not significant as well, marital status was rather consistently protective across the literature analysed, while unemployment was a risk factor, but only in bivariate analysis. Again, both living situation and educational level turned out as non-significant in the majority of the papers. Ethnicity was considered, with contrasting results. Older age, being married or, with weak results, being employed or with higher educational level may then be seen as protective factors towards readmission risk. On the contrary, the presence of benefits or disability pension may represent a risk factor.

Diagnosis is the variable most often analysed, but turned out not significant in many papers. Moreover, comparisons between studies are difficult to implement due to different diagnostic populations and the use of different diagnostic classification systems through the literature. Different measures of severity of illness and in particular the global functioning of the patients (often measured by GAF) could be considered in the prevention of readmission. In general, a worse functioning or prognosis or quality of life, may represent risk factors. Few papers considered the perception and attitude of patients or their compliance to treatment, but interestingly no quantitative paper analysed the subjective point of view of patients on admission, apart from Priebe et al. [14].

The existence or the number of previous admissions were both one of the most often analysed variables and the one most consistently associated with readmission risk. In 20 papers on readmission such a relationship was found in all of the multivariate analyses performed: having previous admissions increased the risk of being readmitted, even after adjusting for other confounders. This confirms the results of previous studies in a more comprehensive sample of papers. Only in a few cases the association between previous hospitalisations and readmissions did not result significant. However, in the majority of these cases there could be two main reasons for the lack of association for this particular result: other covariates associated with previous psychiatric admissions were included in the analysis (such as cumulative LoS, age at onset, etc.); or authors had selected a particular sample of “high or low users” patients.

More in general not only being previously admitted, but also a longer duration of illness and, more consistently throughout the literature, previous use of health services (in particular non-hospital contacts and not only psychiatric ones) are typically risk factors.

### The role of admission’s characteristics and clinical events

Among variables on which the clinicians or policy makers could intervene, papers considered admission characteristics, clinical events or treatment during the admission. The main variable considered was length of stay, which was analysed as a predictor in about half of the papers reviewed; in the majority of these, association of LoS with readmission was non-significant. Moreover, the direction was not very consistent in papers where LoS was found significant; in particular, although in the few multivariate cases where it turned out significant it resulted prevalently protective, for early readmission among patients with dementia and in part of bivariate results opposite findings emerged, suggesting that analysis of the relation between LoS and readmission should be considered for given follow-up periods, age and diagnosis groups, to find more robust results for policy makers. As emerged from our review, other relevant aspects of the discharge process, such as type of discharge, discharge plan and referral made at discharge, have been studied only in very isolated studies.

### The role of contextual factors

Among contextual factors, different types of variables resulted as associated to readmission risk, but were only analysed in some papers and results were not consistent. Deeper analysis of the system level variables has been conducted in another review of the project [10].

A general protective role for social support and caregivers’ positive involvement in care emerged in the review, although different variables were analysed and not always significant results emerged. This result is relevant for policy makers and clinicians and highlights the need for interventions to improve caregivers’ support for treatment (see, e.g., Prince [35]). The protective role of social support seems strengthened also by the fact that being married, employed and with higher quality of social life satisfaction were found associated with a reduced risk to be readmitted.

### Strengths and limitations

Some limitations of this systematic review have to be considered. First of all, the found associations were not straightforward, and the interactions between factors (such as variables related to patients’ severity of illness) complicate the examination of the specific effects of each variable. Another limitation, as described in the quality assessment section, is the low representativeness of the general psychiatric population in the papers as, for example, the authors focused on a specific diagnosis or care program (e.g., substance abuse), or focused only on a specific gender or age group. The main aim of this review was not to provide detailed estimations of overall average effects or associations of each variable with readmission, but rather to give an extensive overview of the studies in this area. Conducting e.g., meta-analysis was deemed unfeasible due to the large number of variables included and the relatively small number examining each of them. In our examination, contrasting results emerged for most variables for different reasons, and we consider the mixed results found as something to be expected due the heterogeneity of settings of the studies. Further reviews could stratify results for severity scores (e.g., using homogeneous samples according to diagnosis, functioning or clinical history) in order to reduce heterogeneity or restrict the included results according to specific follow-up times (i.e., early readmission such as 30 days).

The high heterogeneity of the studies is also due to fact that the studies examined psychiatric populations from different countries, as well as different types of inpatient services and health systems. Some of the differences among the studies are likely to be due to different historical periods (e.g., studies conducted in the 90’s are different from the most recent ones), and to different mental health systems (as countries where the balance between hospital and community is different are expected to show different readmission rates and different influencing factors). This holds within the European countries as well as when Europe is compared for example with the USA. Moreover, the variables examined varied largely among studies; for example, many sociodemographic and clinical variables were categorised differently in different studies, making their comparison a complex exercise.

Moreover, we should consider that, in this review, only “readmission rates” studies have been included while studies and analyses on “time to readmission” and on “heavy/frequent users” were excluded. Nevertheless, there are also differences regarding the outcome variables used in the reviewed studies; in particular with respect to the time considered, heterogeneity across the reviewed studies also emerged: while some in fact calculate measures of “early readmission”, other use longer periods to assess readmission.

Regarding another quality aspect, almost all studies used multivariate analytical methods, controlling for confounders of the association between predictors and readmission. However, we noted that only around 60% of the studies adjusted for the number of previous hospitalisations, which is the variable most consistently found to be associated with readmission in the literature. We have reported here also some results of bivariate analyses, and many of the variables resulted statistically significant at this level. This aspect should be considered as it highlights that in many cases variables are only spuriously associated with readmission, i.e., they are related with it due to their link with other factors. For this reason, we have used this quality criterion to describe the results, highlighting the results emerging especially in multivariate analyses, when they resulted significant, and separating them from the ones retrieved from the bivariate analyses. Moreover, the number of papers reporting significant results on the number of papers analysing each category of variables has been reported in tables, in order to make the reader aware about the strength of the evidence and about the gaps existing in the literature.

## Conclusions

This systematic review examined pre-discharge factors as predictors of readmission among psychiatric patients. The review identified a vast number of factors that were examined in previous quantitative studies. Those factors are related to patients’ characteristics - demographic, social, economic and clinical aspects and patient’s attitude and perception - to environmental, social and hospital characteristics and to admission and discharge characteristics.

The prevention of unnecessary admissions has an impact on patients and caregivers - avoiding interruption in their lives and work activities-, and also on health expenditure, as admissions are the most relevant component of mental health budgets. The results of this review may contribute to increase knowledge on pre-discharge factors that could be considered by policy-makers and clinicians to predict and prevent readmissions of psychiatric patients. Further studies could also aim to identify readmission risk scores or best models considering all the variables resulted significant in this review.

The review gives an overview not only of the main studied variables, confirming that the most consistently significant predictor of readmissions was previous hospitalisation, but also of less frequently studied aspects. The results suggest that there are some other policies and clinically relevant aspects associated with readmission, including the presence of social and carers support and patient’s positive attitude or satisfaction with treatment. On the discharge factors, for example, discharge planning has received much attention over the past few years within the hospital process to reduce readmission and improve continuity of care [70, 71]. However, some of these findings are based on very few studies, and need to be further explored in new studies.

## Additional files

Additional file 1: Detailed search strategies for a systematic review on pre-discharge factors and psychiatric readmission. (DOC 38 kb)
Additional file 2: Evaluation table describing the study population, the follow up period and the pre-discharge variables analysed and resulted significant. (DOC 197 kb)

## Abbreviations

EL: Eva Lassemo
FA: Francesco Amaddeo
FT: Federico Tedeschi
KW: Kristian Wahlbeck
LS: Liljana Sprah
PH: Peija Haaramo
RS: Raluca Sfectu
VD: Valeria Donisi
