# Supplementary material for: Pre-discharge factors predicting readmissions of psychiatric patients: a systematic review of the literature
Source: BMC Psychiatry. 2016 Dec 16;16:449. doi: 10.1186/s12888-016-1114-0 (PMC5162092; doi:10.1186/s12888-016-1114-0)
Supplement: Additional file 2: — Evaluation table describing the study population, the follow up period and the pre-discharge variables analysed and resulted significant. (DOC 197 kb) [file 12888_2016_1114_MOESM2_ESM.doc]

Additional file 2. Evaluation table describing the study population, the follow up period and the pre-discharge variables analysed and resulted significant

| **Author (first), year** | **Country** | **Study population** | **Follow up period**  (d=day, m=month, y=year) | **Pre-discharge predictors analysed** | **Significant results ***  (sign in parenthesis after variable indicate association with readmission) |
| --- | --- | --- | --- | --- | --- |
| **Observational studies** | | | | | |
| **Lyons, J. S.,1997 [7]** | USA | Patients consecutively admitted to the seven most highly utilized hospitals (provided written informed consent)  Mean age 31  N=255 | 30 d, 6m (bivariate)1 y (multivariate) | Age, gender, diagnosis, hospital, dimensions of the Severity of Psychiatric Illness measure, severity of symptoms, family involvement, substance abuse complications, suicide potential, pre-morbid level of dysfunction, Acuity of Psychiatric Illness scale at admission and discharge (and difference), LoS | Bivariate:  30d: impairment in self-care (+)  6m: higher clinical status scores (+), higher level of severity of symptoms (+), more family involvement (-)  Multivariate:  impairment in self-care (+), severity of symptoms and premorbid level of dysfunction (+); suicide potential (-) |
| **Priebe, S. 2009 [14]** | UK | Patients admitted under Sections 2, 3 and 4 of the Mental Health Act 1983  or voluntarily admitted but subsequently detained under these sections within 7 days of admission;  All psychiatric diagnosis included  Mean age 39.5  N=1570 | 1 y | Age, gender, ethnicity (just for adjustment for interviews), diagnosis, legal status; past hospitalization and use of services; perceived coercion rated by patients at admission; perceived risk to self and others; patients’ satisfaction with treatment; living alone vs with others | Bivariate: no significant association for whole sample;  only interviewed at follow-up: being in receipt of welfare benefits (+); living alone, past hospitalisation, satisfaction with treatment (-)  Multivariate (only reinterviewed):  patients receiving welfare benefits (+),higher satisfaction with their treatment at baseline (-), living alone (-) |
| **Craig, T.J. 2000 [17]** | USA | First admitted in 12 psychiatric facilities, presenting clinical evidence of psychosis (schizophrenia and schizoaffective disorders, bipolar disorder with psychotic features, major depression with psychosis features) N=402 | 3m, 1 y (early vs late readmitted; readmitted vs not only for diagnosis) | Gender, age, marital status, ethnicity, diagnosis, medication prescription, presence of symptoms at discharge, type of hospital (university, public, community) | Bivariate:  Diagnosis: Psychotic diagnosis (+), schizophrenic diagnosis (-) (but diagnosis not significant in readmitted vs not); across all diagnoses: having any active symptomatology and affective symptoms at discharge (+); schizophrenic/schizoaffective disorder: presence of psychotic symptoms (+); bipolar disorder: receiving mood stabilizers at discharge (-); depressive psychosis: receiving antipsychotic medications (-)  Multivariate not performed |
| **Ono Y. 2011 [18]** | Japan | First hospitalization  Patients with dementia  Mean age 81  N=326 | 3m, 2y (early vs late vs not readmitted) | Gender (bivariate analysis), age, diagnosis, initial and final scores of: HDS-R, ASSD (Assessment Scale of Symptoms of Dementia), N-ADL (cognitive, behavioural and functional status); complications during hospitalization, care distress and identity of Caregiver, number of cohabitants, length of stay | Bivariate:  3-group comparison: women (late)  A) men: low cognitive function (late), small number of cohabitants at home (late); complications during hospitalization (early); final ASSD (+).  B) Women: complications (early), high LoS (early); high cognitive and physical function (late), short LoS (late), caregivers distress(late)  Multivariate:  For early vs no readmission:  A) Men: number of cohabitants at home (+),  B) Women: higher LoS (+), For late vs no readmission (only performed for women): initial and final score on N-ADL (+), caregivers distress (+); higher LoS (-) |
| **Morrow-Howell, N.L.2006 [19]** | USA | Older patients with major depression hospitalized in geropsychiatric unit  Mean age 76  N=199 | 30 d (rapid readmission), 6 m (readmission vs nursing vs community) | Age, gender, race, education, income, Medicaid, marital status, social support, living arrangement, rural/urban, late onset depression, first episode, number of previous psych hospitalizations, admission values and changes in: BPRS, GAF, GDS, MMSE;, psychosocial problems, inpatients LoS, ECT, | Bivariate:  Rapid readmission: number of psychosocial problems at discharge (+), receiving ECT in the hospital stay (-), longer LoS (-); B) Three-categories: lower GAF score at admission and discharge (nursing),  Multivariate (Three-categories):  lower GAF score at discharge (nursing; GAF at admission excluded due to collinearity with GAF at discharge) |
| **Phibbs, C. S. 1997 [24]** | USA | VA inpatient discharges from substance abuse (including dual diagnosis)  Mean age 42  N= 50,352 | 30, 90, 180 d and 1 y (results reported only for 180 days because similar in other periods) | Number of previous admissions, age, category C eligibility or non-veteran vs lower income veteran, married vs not, black vs not, psychiatric comorbidities: schizophrenia/bipolar/PTSD; depression/borderline/other psychiatric; service-connected disability; type of substance abuse: alcohol, drug, cannabis, nicotine or amphetamine, barbiturate, cocaine and PTSD interaction | Bivariate not performed  Multivariate:  Age (quadratic effect); service-connected disability (+), not married (+), drug (+), barbiturate (+), schizophrenia/bipolar/PTSD (+), depression/borderline/other psychiatric (+), cocaine and PTSD interaction (+), higher number of previous admissions (+); category C or non-veteran (-), black (-), cannabis (-), nicotine or amphetamine (-) |
| **Bowersox, N.W. 2012 [25]** | USA | Veterans with three or more lifetime psychiatric hospitalizations receiving care within an inpatient psychiatric ward  Male 93.6%  Mean age 50  N=233 | 2 y | Age, race, gender, marital status, educational level, monthly income, diagnosis, status (whether they had been assigned a mood, psychotic, substance dependence, anxiety, axis II, or serious medical condition diagnosis - e.g., diabetes, congestive heart failure), treatment history (inpatient days, length of illness, number of lifetime psychiatric inpatient treatments) and illness severity (GAF scores during the previous year of treatment), levels of perceived treatment support from significant others, level of intent to attend outpatient medical and psychiatric appointments | Bivariate:  inpatient psychiatric days during the prior year (+), number of lifetime psychiatric inpatient treatments (+), length of course of illness (+), level of intent to attend outpatient medical and psychiatric appointments (+); being married (-), GAF score: at admission (-) and in the year prior to the study (lowest value) (-), levels of perceived treatment support from significant others (+)  Multivariate:  number of inpatient days during the prior year (+), number of lifetime psychiatric admissions (+) |
| **Dausey, D. J. 2002 [27]** | USA | Veterans psychiatric inpatients who had been discharged from VA Medical Centers  Mean age 50  Male 94.5%  N=35782 | 14, 30 and 180 d | Receipt of preadmission care (as continuous, binary and categorical variable) | Bivariate:  preadmission care as a binary variable (30, 180 d), number of preadmission care visits as a continuous variable (14, 30, 180 d) and as a smooth function (30, 180 d; only for more than 12 visits for 14 days) (+)  Multivariate not performed |
| **Zeff, K. N. 1990 [28]** | USA | All active duty patients admitted to the psychiatric service  Male 87%  Mean age 25  N=246 | 90 d | Axis I diagnosis: severe (mood, psychotic, somatoform and dissociative disorders) vs mild or moderate (adjustment disorders, drug/alcohol abuse/dependence); presence or absence of Axis II diagnosis; LoS, age, gender, years of (active duty: military) service, branch of service, military rank, marital status, number of children, past psychiatric admissions, whether any medication was prescribed at discharge | Bivariate:  severe Axis I disorder (+), presence of Axis II disorder (+), longer LoS (+)  Multivariate not performed |
| **Stahler, G.J. 2009 [32]** | USA | Inpatients with a diagnosis of co-occurring disorders (at least one mental disorder and a substance use disorder)  Mean age 38  N=380 | 1 y | Demographic characteristics (age, race, gender), psychiatric assessment information, drug use data, ; neighbourhood environment variables, related to neighbourhood social disorganization: 1) indicators of socioeconomic disadvantage, 2) community isolation as a result of language barriers, and 3) social disorder as embodied by the presence of crime and dilapidation of housing infrastructure; indicators of alcohol and drug availability: 1) locations of drug possession arrests and 2) establishments licensed to sell beer and liquor under the Pennsylvania State Liquor Control Board; distance from relevant locations; percentage of people with age 25+ with high school or equivalent | Multivariate:  Hispanic patients (+), at least one prior hospital admission (+), living in close proximity to a Narcotics Anonymous meeting location(+); Patients living in areas with higher educational attainment (-), having a chief complaint of depression (-) |
| **Becker, E. A. 2007 [33]** | USA | Adult patients admitted voluntary, with LoS no longer than 1 year  Schizophrenia (comorbidity allowed 49%)  Mean age 40  N=5212 | 5y | Age at time of admission, ethnicity, gender, marital status, county of residence (rural vs metropolitan and whether living in the same county of hospital); diagnosis in comorbidity and number of diagnoses (DSM Axis I and II); LoS, referral to non-hospital based local community MHMR centre for services | Bivariate:  Male (+), Hispanic (+), comorbid personality disorder (+) and alcohol-drug abuse (+), having service assignment in community MH centre (+); older age (-), longer LoS (-), married (-)  Multivariate:  personality disorder (+), Hispanic (+), living in the same county in which the hospital was located (+); longer LoS (-), married (-), older age (-) |
| **Prince, J.D. 2006 [35]** | USA | Newly admitted inpatients enrolled in or eligible for Medicaid with a diagnosis of schizophrenia or schizoaffective  disorder. Excluded patients with severe  and disabling medical condition and LoS>120 days  Age range 17-65  N=264 | 3m | Age, gender, race, marital status, education, prehospital location, paid employment or not  , number of previous hospitalizations, whether admission was involuntary, LoS, clinical symptoms (BPRS, GAS) at discharge, comorbid diagnoses (substance use, alcohol, drug, depression), involvement in care (active engagement and refuse outpatient referral), family factors (criticism, visit unit, meeting with staff, therapy), family psychoeducation, whether family refused involvement, whether patient refused family contact, pharmacological treatment (encouraging of depot; prescription and starting of depot and atypical antipsychotic) | Bivariate:  four or more psychiatric admissions before their index hospitalization (+), substance use (+ ), major depressive disorder (+), refuse at least 1 referral for outpatient care during the inpatient stay (+), family less involved in treatment (+); family involvement (visit, meeting with staff, therapy; psychoeducation: both family not refusing involvement and patient not refusing family contact) (-), lower levels of criticism from family (-), prescription of atypical antipsychotic (risperidone and clozapine) (-)  Multivariate not performed |
| **Warnke I. 2010 [36]** | Germany | Psychiatric inpatient with Schizophrenia  Mean age 35  N=103 | 1y | Age, gender, nationality, married or not, living alone or not, low (no degree) vs high education, job or not, early vs late onset, comorbidity with substance abuse or not, number of di previous admissions, LoS | Bivariate:  low education (+), number of previous admissions (+); LoS (-)  Multivariate not performed |
| **Downing A. 1999 [37]** | UK | Service users discharged from a psychiatric unit of a district general hospital with a care plan. All but one have a diagnosis of psychosis  Age range 18-70  N=35 | 6m | Duration of illness, diagnosis, legal status of service users (involuntary or not), living alone or not, LoS, number of previous admissions (more vs at most 2), attendance of a carer at the discharge meeting or not | Bivariate:  more than 2 previous admissions (+), attendance of a carer at the discharge meeting (-)  Multivariate not performed |
| **Cummings S.M. 1999 [40]** | USA | All patients from the neuropsychiatry unit of a geriatric hospital with a diagnosis of dementia  N=131 | 1m | Gender, age, marital status, LoS, admitting diagnosis, number of diagnoses; relationship of caregiver, presence of: problematic behaviour , heavy care, family conflict, caregiver’s denial of: diagnosis, prognosis and functional impairment status, insufficient support system, caregiver's over-realistic estimation of their own ability to provide car, discharge to nurse or personal care home with no units specialized on dementia, unaffordability and unavailability of the resources required by the patient to meet their needs, social worker adequacy rating of discharge plan | Bivariate:  patient requiring heavy care (+), denial of prognosis (+), insufficient caregiver support system (+), unrealistic caregiver's estimation about own ability to provide care (+), denial of the patient’s level of functional impairment (+), male (+); higher social worker adequacy rating of discharge plan (-)  Multivariate:  male (+), insufficient caregiver support system (+); higher social worker adequacy ratings of discharge plan (-) |
| **O'Donoghue, B. 2011 [41]** | Ireland | Patients admitted involuntarily  Mean age 40  N=90 | 1y | All patients: gender, admission source (public vs private catchment area), Marital status, Diagnosis (schizophrenia/schizoaffective disorder, affective disorder, other), whether or not: first admission, coercion. Reinterviewed: the MacArthur Admission Experience Interview,  Birchwood Insight Scale, the Drug Attitude Inventory, GAF  and the Recovery Style Questionnaire | Bivariate:  A) Total sample: admitted from the public catchment area (+); first admission to hospital (-).  B) Reinterviewed: individuals who have a sealing over recovery style (+)  Multivariate not performed |
| **Walker, R. 1996 [43]** | USA | All adult admissions in voluntary psychiatric unit  Mean age 45  N=423 | 6 m | Only GAF score at admission, gender and race considered for readmission as a binary outcome | Bivariate:  higher GAF-A scores at index admission (-)  Multivariate:  Not performed for readmission as a binary outcome |
| **Gunnell, D. 2008 [44]** | UK | Patients admitted  to general adult psychiatry inpatient beds  Age range 16-24  N=75401 | 1y | Gender and (for diagnostic subgroups) LoS only | Bivariate:  Gender: female (+). Length of stay: for all diagnoses apart from organic disorders (NS): longer LoS (-), even after controlling for age and gender  Multivariate not performed |
| **Clements, K. M. 2006 [46]** | USA | Inpatients admitted to a psychiatric hospital for which a BASIS-32 was completed at both admission and discharge  Age 18+(13% 51+)  Age N=1034 | 1y | Both multivariate and bivariate: GAF and BASIS-32 (other significant variables just controlled). Only bivariate: diagnostic codes for Axis I, Axis II (personality disorder) of DSM-IV, age, gender, race, marital status, employment/student status, insurance coverage, admission and discharge dates, hospital unit (short stay vs mood disorders unit) | Bivariate:  employed or students (-), previous admissions (+), higher admission BASIS-32 score (+), longer LoS (+), hospital unit (not specified how), mood and schizophrenic disorders (+), substance abuse and other disorders (-)  Multivariate:  BASIS-32 score (+) |
| **Wong, Y. C. 2006 [48]** | China | Patients first conditionally discharged with severe mental illness  Mean age 39  N=140 | 1y | Gender, Age, Age of psychiatric illness onset, Immigrant status, Marital status (Single; Married / cohabiting; Widowed / divorced), Living status (Alone, With family, With others), Accommodation (Public housing; Private housing), Education (Primary or less, Secondary, Tertiary), Income (Self, Public assistance), Occupation (Working, Unemployed, Housewife, Student); raised by the social welfare service/in foster care vs by parents/relatives;  Forensic history; History of substance misuse; Violence history; Suicide attempt or deliberate self-harm history; family history of suicide; history of  physical disease(s); Psychiatric illness before index admission; whether there were previous psychiatric admissions;  number of previous admissions; average duration of previous admissions; PFU target; Community Psychiatric Nurse follow-up and Irregular and default OPD follow-up in the pre-discharge period; Poor drug compliance;  Mental illness co-morbidity; duration of no treatment period; Conditionally discharged by Doctor / MDCC MHRT; LoS; Medication at index admission: depot or not, on new anti-psychotic drug or not | Bivariate:  single (+), raised by the social welfare service/in foster care (+), family history of suicide (+), both having prior psychiatric admissions and their frequency (+), having mental illness comorbidities (+); older age (both at index date and at illness onset) (-), violence history (-)  Multivariate:  number of psychiatric admissions (+), raised by the social welfare service/in foster care (+); older age at index date (-) |
| **Hendryx, M. S. 2001 [49]** | USA | Adults discharged from inpatient psychiatric care to a designated community mental health centre having at least 30 referral clients  Mean age 38  N=1616 | 60d (readmission considered from day 14) | Age, gender, marital status, race and Hispanic ethnicity, residential status, employment status, DSM-IV diagnoses and Axis I-Axis V values, SMI ratings, GAF scores, legal status (court involuntary, order of detention or voluntary) | Bivariate:  age 38+ (-), court involuntary admission (-)Multivariate:  age 38+ (-), court involuntary admission (-); Axis IV economic problems (+); Black (+) |
| **Hendryx, M. S. 2003 [50]** | USA | Patients with psychiatric hospitalization  Mean age 38  N=1384 | 1y | Bivariate: diagnosis. Multivariate:  age, gender, race (white vs non-white), outpatient hours in year before index hospitalization, previous State and Harborview hospitalization or not, housing status (homeless, living alone, other), married or not, employed or not, and diagnosis severity score, GAF at admission, secondary or tertiary substance abuse or not, BPRS, suicide risk score, assaultive behaviour history or not, denial of chemical dependency at discharge, denial of psychiatric illness at discharge, involuntary index admission or not, index discharge against medical advice or not, alcohol or drug problem severity rating; activity of daily living, role dysfunction, unreliability of discharge social support | Bivariate:  Ranking among diagnostic groups: schizophrenia, other diagnosis, bipolar disorder, depression  Multivariate:  prior State hospitalization and prior outpatient use (+) ; activity of daily living dysfunction and social support unreliability (+); higher age (-), GAF (-), secondary or tertiary substance abuse (-) |
| **Grinshpoon, A. 2007 [51]** | Israel | Discharged from their ﬁrst-in-life psychiatric hospitalization in 1990–1991 or in 2000– 2001 | 3y | Bivariate: 1991 vs 2001 cohort (globally and for LoS below and above 6 months, respectively). Multivariate: gender, age, marital status, whether Jewish or not  demographic variables + complex variable—‘‘cohort/length of hospitalization’’ diagnosis only used as a mediator variable | Bivariate:  2000-2001 vs 1990-1991 (-) (both globally and for LoS longer than 6 months, NS for LoS < 6 months)  Multivariate:  Among affective: divorced (vs single)(+); among schizophrenics: 2000-2001 (but only for LoS > 6 months)(-); higher age (-) |
| **Mark, T. 2013 [52]** | USA | Only hospital with at least 25 Medicaid admission with M/SUD diagnosis. Patients were required to have continuous health plan enrolment for at least 180 days prior to the index admission and for 30 days following discharge.  Mean age 28  N=121271 | 30d (readmission considered from day 8) | Patient level: Age, race, gender, urban or rural residence,  enrolment in a fee-for-service or capitated Medicaid plan; psychiatric principal diagnosis on the  index admission (AHRQ MH clinical classification); hospital-level: Annual number of Medicaid stays with a principal M/SUD diagnosis, median LoS for M/SUD Medicaid discharges, index admission inpatient procedures: psychological evaluation and testing; psychiatric interviews, consultations and evaluations; psychiatric somatotherapy; other psychotherapy and counselling; alcohol and drug rehabilitation and detoxification | Bivariate:  Hospital level: number of Medicaid patients with M/SUD (+), interviews, consultations and evaluations, somatotherapy, individual psychotherapy (+); other psychotherapy and counselling, alcohol and drug rehabilitation and detoxification (-); median LoS for Medicaid patients with M/SUD (-); Patient level: age between 18 and 54 (+), male (+), African American (+), a fee-for-service Medicaid plan (+), urban area (+);having schizophrenia or other psychotic disorders (+); adjustment, anxiety, attention deficit, miscellaneous mental and mood disorder (-); use of medication and behavioural health services in the 6 months prior to the index hospitalization: prior M/SUD admission (+), having an outpatient M/SUD visit or visiting a psychiatrist (+),prescription fill of: antipsychotic medication and substance use (+)  Multivariate:  prior admission for M/SUD (+), index admission diagnosis of schizophrenia or another psychosis and of mood disorder (+), in the 6 months prior to an index admission: medication fill for a substance use disorder, antipsychotic fill (+), having an outpatient visit for M/SUD and for CMHC (+); living in an urban setting (+), having a substance abuse disorder pre-index admission (+), having hypertension (+), being male (+); being Hispanic (-); hospital level: annual mean stays of patients with M/SUD diagnosis (+); hospital level: median LoS (-) |
| **Zhou Y. 2014 [53]** | China | Any discharge in the study period with a primary psychiatric ICD-10 diagnosis  Mean age 42  N=2525 | 1y | Age, gender, diagnosis, LoS, number of previous admissions (0, 1, 2, 3+) | Bivariate:  longer LoS (+), number of previous admissions (+), age 41-50 and <20 (-)  Multivariate not performed |
| **Carr, V. J. 2008 [54]** | Australia | Admission in acute psychiatric inpatient units  Mean age 38  N= 5546 admissions | 28d | Both bivariate and multivariate: Gender, Age, Marital status, Past psychiatric admissions (both at least one ever and within 28 days of index one).  Legal status: Involuntary or not, discharge diagnosis (yes or no for each diagnosis: schizophrenia, bipolar, depression, adjustment, personality, drug/alcohol, physical)  Only bivariate: LoS, Reportable aggressive incidents, Less serious aggressive incidents, Absconding incidents | Bivariate:  Reportable aggressive incidents (+), past admissions and previous admissions within the last 28 days (+), personality disorder (+), depression (+); female (-), adjustment (-)  Multivariate:  reportable aggressive incidents (+), past admissions and previous admissions within the last 28 days (+), personality disorder (+); LoS (-),female (-) |
| **Sanchez, R. 2013 [55]** | Colombia | All patients admitted to a psychiatric hospital  Mean age 40  N=214 | 1m, 3m | Gender, age, education, social support, socio-economic status, LoS, number of previous episodes of illness (including those without hospitalization), length of illness, diagnosis (following DSM Axis I and II), use of substances, type of discharge (on medical advice, against medical advice, referred to other centres due to temporary remission of symptom) | Bivariate not performed  Multivariate:  30 days: single (vs married) (-), male gender (-); 90 days: referral to other centres due to remission (vs discharged on medical advice) (+), substance use (+), separated/divorced (vs married) (+); secondary diagnosis (being primary diagnosis a medical condition) vs bipolar disorder (-) |
| **Kottsieper, P. 2006 [56]** | USA | Individuals with serious mental illness discharged from an acute inpatient stay (several inclusion and exclusion criteria)  Mean age 39  N=74 | 3m | Gender, race, insurance (Medicaid or not), marital status (single or not), discharge diagnosis (schizophrenia or not), current substance use or not, main treatment problem (MH, dual, substance abuse, neither), past aftercare adherence or not, housing at discharge, referred where (clinic vs walk-in), age, los, number of previous hospitalizations, Motivational Beliefs questionnaires (on treatment motivation, attitude towards psychiatric medication, reasons for entering treatment and  their feelings about treatment) | Bivariate:  number of prior hospitalizations (+), discharge diagnosis of schizophrenia spectrum disorders (vs bipolar and depression spectrum disorders)(+); no insurance vs Medicaid (-), positive attitude towards psychiatric medication(-)  Multivariate:  number of prior hospitalizations (+); schizophrenia spectrum disorders (vs bipolar and depression spectrum disorders) (+); positive attitude towards psychiatric medication (-); male(-) |
|  |  |  |  |  |  |
| **Bernardo, A.C. 2001 [58]** | Canada | Random selection among all discharges from psychiatric hospital  Man age 37  N=200 | 3y | Number of admissions (both to the study hospital and to any psychiatric facility) before the index admission, marital status, education, employment status, diagnosis, history of aggression and of behavioural problems, history of suicide attempts or physical, sexual, or emotional abuse | Bivariate:  number of previous admissions (both to the study hospital and to any psychiatric facility) (+), divorced (+), secondary school education (+), unemployed or employed only part-time or receiving social assistance (+); a history of aggression (+) and of behavioural problems (+); primary education or vocational or technical school (-); diagnosis (but not specified how)  Multivariate not performed |
| **Russo, J. 1997 [59]** | USA | Severely  ill and largely indigent patients admitted  to either locked or voluntary  inpatient units  Mean age 36  N=1053 | 18m | Age, gender, ethnicity; unit the patient was on, whether s/he was involuntarily committed; primary psychiatric diagnosis, history of psychiatric hospitalization, presence of a comorbid substance use disorder, admission 23-item BPRS scores on (PSAS), and patients’ level of insight into their psychiatric illness at admission; 13 functional QOLI indexes; 7 scores of life satisfaction (global, living arrangements, family relations, social relations, leisure activities, personal safety, finance) | Bivariate:  previous psychiatric hospitalization (+), comorbid substance use disorder (+), homelessness (+); satisfaction with life at discharge (-), satisfaction with life: global and single scales (-), involuntary committed, locked unit (-), admission PSAS score (-), better insight into illness at admission and at discharge (+), average number of social contacts, frequency of visits with family and with friends, frequency of contacts by phone with family (-),  Multivariate:  locked unit (-), previous psychiatric hospitalization (+), comorbid DSM-IV substance use disorder (+), better insight into own psychiatric illness at admission (+), more contact with family and with friends (-), global life satisfaction reported at admission (-) |
| **Adams, J.D. 2010 [60]** | USA | Discharge from Alaska Psychiatric Institute  Psychotic or mood disorder  Mean age 38  N=915 | 30d | Age, race, gender, marital status (not married, married, widowed, divorced), education (low, medium or high level), employed or not, LoS, number of preadmissions, primary psychiatric diagnosis(psychotic vs mood disorder), discharge living arrangement (alone, house with others, hospital, homeless, homeless shelter, supervised care, jail) and discharge regional code (Southcentral Alaska vs other) | Bivariate:  Unemployed (+), number of preadmissions (+), being discharged to Southcentral Alaska (vs Southcentral Alaska) (+); living alone (-).  Multivariate:  number of preadmissions (+), being discharged to Southcentral Alaska(+); living alone (-) |
| **Owen, C. 1997 [62]** | Australia | All consecutive patients from acute psychiatric services with signed consent  Mean age 35  N=128 | 6m | Gender, age, marital status, working status and best ever employment (skilled work, unskilled work, never worked), ethnicity, residing in the catchment area for 5+ years, housing status, changing accommodation in the last 2 years or not, diagnosis, level of symptoms (BPRS) or functioning (SBS subscales) at discharge, patient attitude to follow-up or "likeability", financial means, prediction at discharge of follow-up compliance, history of previous psychiatric admissions | Bivariate:  more difficulties with hygiene and fewer leisure pursuits (Social Behavioural Schedule) (+); prediction at discharge of follow-up compliance (-), higher financial means (-)  Multivariate:  higher financial means (-) |
| **Thompson, E.E. 2003 [63]** | USA | Patients who had received inpatient care  at a state psychiatric hospital  Mean age 37  N=1,481 | 6m | Gender, race (white versus other), education (less than 12 years of school versus other), age, diagnosis, prognosis (poor, guarded, unknown vs fair or good), LoS, number of previous admissions, informal emotional support or not | Bivariate not performed  Multivariate:  schizoaffective disorder (vs other schizophrenic disorders)(+), higher number of previous admissions (+), a poor prognosis (+) |
| **Ng, C.G., 2012 [66]** | Malaysia | All conservative patients who were discharged from the psychiatric ward  Patients who were diagnosed with any mental illness and consented to be recruited  Mean age 39  N=202 | 6m | Age (above or below 40), gender, race (Malay or not), marital status (never married or not), education (less than vs at least secondary), employed or unemployed, previous life events or not (Life Events Questionnaire); first onset or not, diagnosis (psychotic disorder or not), previous admissions or not, LoS (at least vs below 15 days), atypical (vs conventional, mixed or nil) antipsychotic , depot injectable antipsychotic or not, electroconvulsive therapy or not, substance use or not; Multidimensional Scale of Perceived Social Support and Brief Psychiatric Rating Scale scores  BPRS score, both as a continuous and a binary variable (other variables just controlled for) | Bivariate:  psychotic disorder (+), previous admission (+),on depot injectable antipsychotic (+), higher BPRS scores (+), first onset (-), on atypical antipsychotic (-)  Multivariate:  BPRS score, both as a continuous and a binary variable (+) |
| **Averill, P. M 2001 [69]** | USA | Random sample of inpatients admitted to a hospital providing brief psychiatric services to individuals with serious mental illness  Mean age 36  N=131 | 9m | Number of previous admissions; Brief Psychiatric Rating Scale-Anchored (BPRS-A); The Symptom Checklist 90 Revised (SCL-90-R), Beck Depression Inventory (BDI), Michigan Alcoholism Screening Test (MAST), Drug Abuse Screening Test (DAST); C) Intellectual Functioning: Kaufman Brief Intelligence Test (K-BIT); age, gender, marital status, race, and living environment | Bivariate not performed  Multivariate:  higher self-reported anxiety symptoms (SCL-90-R anxiety scale) (+) and number of previous admissions (+) |
| **Wheeler, A. 2011 [72]** | New Zealand | All admissions for psychiatric reasons from acute hospital services  Mean age 36  N=924 | 5y | Primary diagnosis (categorised as schizophrenia [including schizoaffective disorder], bipolar disorder, depression and other disorders), previous psychiatric admission or not | Bivariate:  no previous psychiatric admission (-); bipolar disorder (+). Ranking among diagnostic groups: bipolar disorder, schizophrenia/schizoaffective, "other" and depression)  Multivariate not performed |
| **Case-control studies** | | | | | |
| **Callaly, T. 2011 [9]** | Australia | Any admission in adult acute mental health inpatient services  readmitted within 28 days VS patients not readmitted during the same period  Age nr (adult)  N=475 | 28 d | Age, gender, employment status, accommodation type, whether on the Disability Support Pension, criminal justice involvement in 6 months prior to index admission, extent of a social support network, age at first receipt of mental health care, diagnosis, whether personality disorder was present, whether the patient was a new or existing consumer for that service, number of admissions in the previous 12 months, LoS, legal status, admission and discharge Health of the Nation Outcome Scales (HoNOS, only for bivariate analyses), whether there were pre-discharge contacts with family or nongovernment psychosocial support organizations (NGOs), being unable to locate a documented discharge plan, record of a plan sent to the GP | Bivariate:  female gender (+), known to the service (+), admissions in the previous 12 months (+), a diagnosis of emotionally unstable personality disorder (+), follow-up care planned (at discharge) to be with the local AMHS (+), being unable to locate a documented discharge plan (+),record of a plan sent to the GP (+); higher HoNOS total score on admission (-)  Multivariate:  female gender (+), known to the service (+), admissions in the previous 12 months (+), a diagnosis of emotionally unstable personality disorder (+), follow-up care planned (at discharge) to be with the local AMHS (+),discharge plan record sent to the GP (+) |
| **Monnelly, E.P. 1997 [16]** | USA | Patients with psychiatric disorder discharged from a VA hospital exclusive of a substance use or cognitive disorder;  Readmitted within 30 days VS non-readmitted in 6 months  Mean age around 46  Male  N=531 | 30 d (readmitted), 6 m (non-readmitted) | A) Admission variables: age, number of hospitalizations in the previous two years, marital status, GAF rating at admission, whether treatment goals were adequately documented in the chart at admission. B) Discharge variables: diagnosis (only multivariate analysis), extent to which treatment goals were met, length of stay, GAF rating at discharge, instability in the three to five days before discharge (i.e.: use of restraints, use of seclusion, requiring orders for close observation in the three days before discharge, active psychotic behaviour, suicide attempts or gesture, assault within five days of discharge, receiving p.r.n. medications - not including hypnotics - or not) | Bivariate:  hospitalizations in the previous two years (+), severe GAF at discharge (+), at least one sign of instability in the three to five days before discharge and not recovering from it before discharge and, separately: receiving p.r.n. medications, use of restraints, use of seclusion, requiring orders for close observation in the three days before discharge, active psychotic behaviour (+); older age (-)  Multivariate:  number of hospitalizations in the previous two years (+), at least 1 sign of instability (+); older age (-) |
| **Touch Mercer, G. 1999 [20]** | USA | Geropyschiatry inpatients  Case-control study (cases matched based on demographic variables)  Male  Mean age 71  N=150 | 18 m | LoS, living arrangements at discharge, diagnosis, GAF score at discharge and at admission, discharge score and change of BPRS, Hamilton Rating Scale for Depression, Cohen-Mansfield Agitation Inventory, substance abuse, number of caregivers (0, 1, 2 or more), Change in the social support network precipitating psychiatric admission, Rating of family or social system functioning | Bivariate:  mood disorder or schizophrenia (+); dementia (-), diagnosis of substance abuse (+)  Multivariate:  Maladaptive family system functioning (+) |
| **Kim, H.M. 2011 [26]** | USA | Patients receiving depression  treatment  Mostly male veterans  Readmitted vs not readmitted  Mean age 51  N= 53363 | 5 y | Age at index discharge; race; Hispanic ethnicity; diagnoses of a substance use disorder, PTSD, major depression, personality disorder, anxiety disorder and bipolar disorder type II; service connection in regard to disability benefits; prior suicide attempt; use of services with Medicare claims; numbers of outpatient visits, outpatient mental health visits, VA psychiatric hospitalizations, LoS, psychiatric inpatient days in previous year, psychotropic medications filled during the year before the index hospitalization facility region and whether the facility  was in an urban area | Bivariate:  number of psychiatric hospital days (+), number of psychiatric hospitalizations (+), more outpatient visits and MH visits in the year prior to index hospitalization (+), being black (+), number of psychotropic medications (+), having longer LoS (+), alcohol and drug disorders (+), facility in the North East (+), suicide attempts (+); disorders: anxiety (-) and depression (-), disability benefits (-), race unknown (-), older age (-)  Multivariate:  being black (+) and from North East (+), number of psychiatric hospitalizations (+) and more outpatient visits in the year prior to index hospitalization (+), at least 3 psychotropic medications (+); risk: age 35-64 (-), 1 or 2 psychotropic medication(-), race unknown (-), disorders : tobacco use (-) and depression (-) |
| **Callaly, T. 2010 [29]** | Australia | Patients admitted in acute psychiatric adult inpatient units  Readmitted vs not readmitted  N=115  Age nr (adult)  Gender nr | 28 d | Number of previous admissions, diagnosis of Emotionally Unstable Personality Disorder, age at onset, ; whether: receiving the Disability Support Pension, discharge plan sent to their GP on discharge from the index admission, having had recent history of substance use or criminal involvement in the 30 days before admission; type of accommodation the patient was discharged to, the 12 item scores of the HoNOS on admission and discharge | Bivariate:  having had an admission in the previous year (+),receipt of the Disability Support Pension (+), unemployed patients (+); discharge plan sent to their GP on discharge from the index admission (-)  Multivariate:  number of previous admissions (+); discharge plan sent to their GP on discharge from the index admission (-) |
| **Dixon, M. 1997 [30]** | UK | Patients discharge from acute psychiatric adult in-patient. Readmitted vs not readmitted  Gender n.r.  Age n.r. adult  N=328 | 6 m | Age, gender, marital status, ethnicity, occupation, living arrangements, number of previous admissions, diagnosis, length of previous hospital stay, number of referrals made on discharge, age at 1st psychiatric admission and whether or not the index discharge was made against medical advice | Bivariate:  living status and number of previous psychiatric admissions (not specified how but likely to be the same as multivariate analysis); discharge against medical advice (+)  Multivariate:  discharge against medical advice (+), higher number of previous psychiatric admissions (+); risk: living in care (vs alone or with family) (-) |
| **Saleh, El-Sayed 2012 [57]** | Egypt and Saudi Arabia | Patients admitted in two psychiatric hospitals  Patients readmitted within 3 months VS patients with only 1 admission in 2 years  Mean age 29  N=300 | 3m (readmission considered from day 8), control group: single admission in 2 y | Age, gender, employment status (skilled worker, unskilled worker and unemployed), marital status, educational level (illiterate, primary school, preparatory or secondary school, faculty education and  post graduate education), rural vs urban residence, duration of illness, LoS, normal vs abnormal (i.e. escape from hospital or discharge against medical advice) discharge, diagnosis, Multidimensional Scale of Perceived  Social Support (MSPSS) and Medication Adherence Rating Scale (MARS) | Bivariate:  male gender (+), unemployed (+), unmarried (+), primary education and illiteracy (+), bipolar or schizophrenia disorders (+), living in rural areas (+), abnormal discharge (+), duration of illness (+), scores on MARS (+), substance use (+); skilled workers (-), faculty education (-), depression or adjustment disorders (-), scores on MSPSS (-), longer LoS (-); personality disorders (-)  Multivariate not performed |
| **Comparative naturalistic studies** | | | | | |
| **Brennan, P. L. et al., 2000 [15]** | USA | Patients aged 65+ discharged from hospital with a substance abuse, dependence or psychosis diagnosis or an ICD-9 surrogate medical diagnosis indicative of alcohol abuse or dependence;  Surviving patients with substance use disorders VS surviving controls out substance use disorders on rates of readmission  N=12,417 | 4 y | Bivariate analysis: study vs control group; gender. In gender-separate multivariate analysis: age, race; prior hospitalization with a substance use disorder, characteristics of the index episode, psychiatric diagnosis, number of psychiatric diagnoses, LoS | Bivariate:  study (vs control) group (+), female gender (+)  Multivariate:  female gender (+)  A) Both women and men: prior hospitalization with a substance use disorder (+), dual psychiatric and substance use disorder diagnoses (+), having more mental health diagnoses (+).  B) Women only: older age (-) |
| **Moos, R. H. 1994 [21]** | USA | Surviving patients' treatment back over the 4 years before the index episode. age 55+  Alcohol and/or drug dependence diagnoses and no other substance abuse or psychiatric diagnosis, diagnosis of alcohol or drug psychos, alcohol or drug dependence and/or alcohol or drug psychoses who also had one or more psychiatric diagnosis  Male> 99%  Mean age 62  N=16,066 | 1y, 4y | Associated alcohol or drug psychosis or psychiatric diagnosis in: index episode, prior episodes; number and length of episodes of inpatient care for substance abuse and/or psychiatric disorder , place of index episode (substance abuse, medical detox, psychiatric units or extended care unit), LoS (up to 14 days vs 14+) and whether the patient's discharge was against medical advice; outpatient visits in the prior year for mental health or medical care (including visits for specialized substance abuse and psychiatric care) and, if so, the number of visits for each type of care, age, gender, race, marital status, presence of a service-connected disability | Bivariate:  presence of a service connected disability (+), psychiatric diagnosis in a prior episode (+); others not mentioned  Multivariate:  Unmarried patients (+), younger age (55-64) (+), number of inpatients episodes (+), 3 or more outpatient medical visits in the 4 years before the index episode (+), psychiatric diagnosis (+), being treated in psychiatric units (+), LoS up to 14 days (+), Alcohol or drug psychosis (+) |
| **Moos, R. H. 1995 [22]** | USA | Substance abuse inpatients (including dual diagnosis)  Mean age 42  Male 99%  N=10352 | 6m, 1y | Gender, age, education, race, presence or absence of alcohol-related or drug-related diagnoses without a psychiatric diagnosis, prior outpatient mental health or medical care, LoS and the number of episodes of inpatient care in which patients had either a substance abuse or a psychiatric diagnosis, or both, or in which they had only a medical diagnosis, in the 4 years before the index episode | Bivariate:  same variables significant in at least one of the multiple regressions, but neither specified how nor significance  Multivariate:  unmarried at intake (+, 1 year only); prior inpatient substance abuse or psychiatric episode (+), prior medical episode (+), psychiatric diagnoses (+) |
| **Moos, R. H. 1995 [23]** | USA | Substance abuse inpatients (including dual diagnosis)  Older VS middle-aged VS younger patients before  Mean age 45  Male ≥ 99%  N= 33,323 | 1 y | Main variable: age (18-34, 35-54, 55+). Other variables: gender, race, marital status, diagnosis; number of episodes of inpatient care in which patients had either an  ICD-9-CM substance abuse diagnosis or an ICD-9-CM psychiatric diagnosis or both, or only a medical diagnosis, during the 4 years before the index episode; whether patients had outpatient visits during the prior year for mental health or medical care and, if so, the number of visits for such care | Bivariate:  A) General: older patients (+); younger patients (-). B) For all age subgroups: longer LoS (-)  Multivariate:  All age-groups: unmarried status (+), more prior inpatient episodes for treatment of substance abuse or psychiatric disorders (+) and medical disorders (+), more prior outpatient mental health (+) and medical care (+), and an alcohol or drug psychosis diagnosis (+) or a psychiatric diagnosis (+). |
| **Schoenbaum, S. C. 1995 [31]** | USA | Psychiatric inpatients Health centres division VS Medical group division  N=580  Gender n.r.  Adults Age n.r. | 6m | Bivariate: index admission to health-centre vs medical group divisions. Multivariate: previous relationship with a mental health practitioner or not, health-centres division, gender, LoS, discharge status (absent without leave or not), diagnosis (adjustment disorder, affective disorder, other) | Bivariate:  health-centres division  Multivariate:  previous relationship with a mental health practitioner (+), health-centres division (+); male gender (-) |
| **Lin, H. C. [34]** | Taiwan | Patients with schizophrenia divided equally into four groups according to LOS of index hospitalization  N=29,373  Age: <40: 56.0%, 40-60: 37.8%, >60: 6.2% | 30d | Also multivariate: LoS groups  Only bivariate:  hospital characteristics (level, ownership, location, teaching status); physician and patient characteristics (gender and age) | Bivariate:  male gender (+), older age (+),younger doctors (+), discharged from regional and public hospitals (+); longer LOS (-), medical centre (-), not-for-profit (-)Northern/Eastern hospital (+)  Multivariate:  longer LOS (-) |
| **Colenda, C. C.1991 [38]** | USA | Depressed patients  late-onset  geriatric Vs early-onset geriatric and young adult  N= 111  Mean age 56 | 2y | Comparison among 3 groups: late onset geriatric depression, early onset geriatric depression, young adult depression | Bivariate:  late onset geriatric depression (+); young adult depression (-)  Multivariate not performed |
| **Kreys, T. J.2013 [39]** | USA | Patients with a bipolar disorder diagnosis at hospital discharge newly initiated on therapy during their inpatient stay  Ariprazole vs quetiapine pharmacological treatments  N=286  Median Age 37-40 | 30d | Main variable: ariprazole vs quetiapine pharmacological treatment  Others: age, gender, race, current bipolar episode diagnosis, psychiatric hospitalizations in the prior year or not, presence of a comorbid substance abuse or dependence diagnosis, concomitant inpatient psychotropic medications, LoS, LoS on the index antipsychotic, discharge dose of the index antipsychotic, concomitant psychotropic medications at discharge | Bivariate:  previous admissions (+)  Multivariate not performed due to only 1 significant variable significant in bivariate analysis |
| **Snowden, M.B. 2004 [47]** | USA | All inpatient psychiatry admissions  Aged 65+ (mean age 74) VS younger (mean age36)  N=5091 | 1y | Age-groups (other variables just controlled for): 65+ vs younger | Bivariate: elderly (+)  Multivariate: no significance |
| **Winston, A. 1997 [73]** | USA | All patient in inpatient community unit in municipal hospital  Receiving aftercare treatment and not treatment at follow up  Age range 10-69, age 10-19 <20%  N=114 | 1y | Main variable not considered because post-discharge.  Diagnosis (schizophrenia vs other diagnoses, schizophrenia vs psychotic depression) | Bivariate: Schizophrenia (vs other diagnoses) (+)Multivariate not performed |
| **Intervention studies** | | | | | |
| **Papageorgiou, A. 2002 [42]** | UK | In-patients receiving compulsory psychiatric  Treatment  Advance  directives vs usual care  N=156  Median age 36 | 1y | Intervention group vs advance  directives vs control group | Bivariate:  Intervention not significant  Multivariate not performed |
| **Kolbasovsky, A.2009 [45]** | USA | Discharge  from an acute hospitalization with a primary psychiatric  diagnosis  Intensive case management VS control group (intention-to-treat, historical control design)  N= 652  Mean age 47 | 30d | Intervention: Intensive case management (ICM) services vs baseline group (patients meeting eligibility criteria prior to implementation). Other variables: age, gender, insurance (commercial, Medicare,  Medicaid), psychiatric diagnosis category (depressive, bipolar,  psychotic, other psychiatric disorder), LoS, predictive model  risk score | Bivariate not performed  Multivariate: baseline group (+); Medicaid insurance coverage(-) |
| **Swartz, M. S. 1999 [61]** | USA | Subjects who were hospitalized involuntarily with diagnosis of schizophrenia, schizoaffective disorder, or  other psychotic disorder or major affective disorder (including several inclusion and exclusion criteria)  Randomly assigned to  be released or to continue under outpatient commitment  Mean age 40  N=129 | 1y | Intervention (both bivariate and multivariate):  released vs continue under outpatient commitment  Other variables (only multivariate): age, race (African American or not), gender, education in years, marital status (single and not cohabiting vs married/cohabiting); diagnosis (non-affective psychotic vs affective disorders), GAF Scale score, Insight and Treatment Attitudes Questionnaire score, presence of at least one source reporting fighting and substance use, medication noncompliance (all measured over the 4 months before the baseline interview). | Bivariate: no significant difference between groups  Multivariate: outpatient commitment group (-), higher GAF 4 months before (-), education (-) , psychotic diagnosis (for outpatient commitment group only) (-) |
|  |  |  |  |  |  |
| **D'Ercole, A. 1997 [74]** | USA | Psychiatric hospital inpatients  Randomly assigned to an outreach case management team or standard aftercare. Mean age 35  N=289 | 18m | Intervention not considered for readmission as a binary outcome.  Gender | Bivariate: no significance difference  Multivariate not performed |
| **Schmidt-Kraepelin, C. 2009 [75]** | Germany | Patient s suffering by schizophrenia or schizoaffective disorder recruited during their inpatient treatment A complex intervention with improved cooperation between in- and outpatient services VS treatment as usual  Mean age 43  N=93 | 1y | Intervention not considered because post-discharge.  Age, gender, family status, level of education, number of previous hospitalizations | Bivariate: no significance  Multivariate not performed |
|  |  |  |  |  |  |

Gender is reported only if the population was predominantly reported as male or female (i.e. > 80%); Mean age is reported if not available other information on age is reported (e.g. range, % of young or 65+); Diagnosis is reported only if a specific diagnostic group is considered. *p-value < 0.05.
